# Chemoenzymatic Synthesis of Keratan Sulfate Oligosaccharides Using UDP-Galactose-6-aldehyde To Control Sulfation at Galactosides

**DOI:** 10.1021/acs.orglett.4c02899

**Published:** 2024-09-23

**Authors:** Yunfei Wu, Gerlof P. Bosman, Gaël M. Vos, Elif Uslu, Digantkumar Chapla, Chin Huang, Kelley W. Moremen, Geert-Jan Boons

**Affiliations:** † Chemical Biology and Drug Discovery, Utrecht Institute for Pharmaceutical Sciences, Utrecht University, Universiteitsweg 99, 3584 CG Utrecht, Netherlands; ‡ Complex Carbohydrate Research Center, University of Georgia, 315 Riverbend Road, Athens, Georgia 30602, United States; § Department of Biochemistry, University of Georgia, Athens, Georgia 30602, United States; ∥ Bijvoet Center for Biomolecular Research, Utrecht University, 3584 CH Utrecht, Netherlands; ⊥ Department of Chemistry, University of Georgia, Athens, Georgia 30602, United States

## Abstract

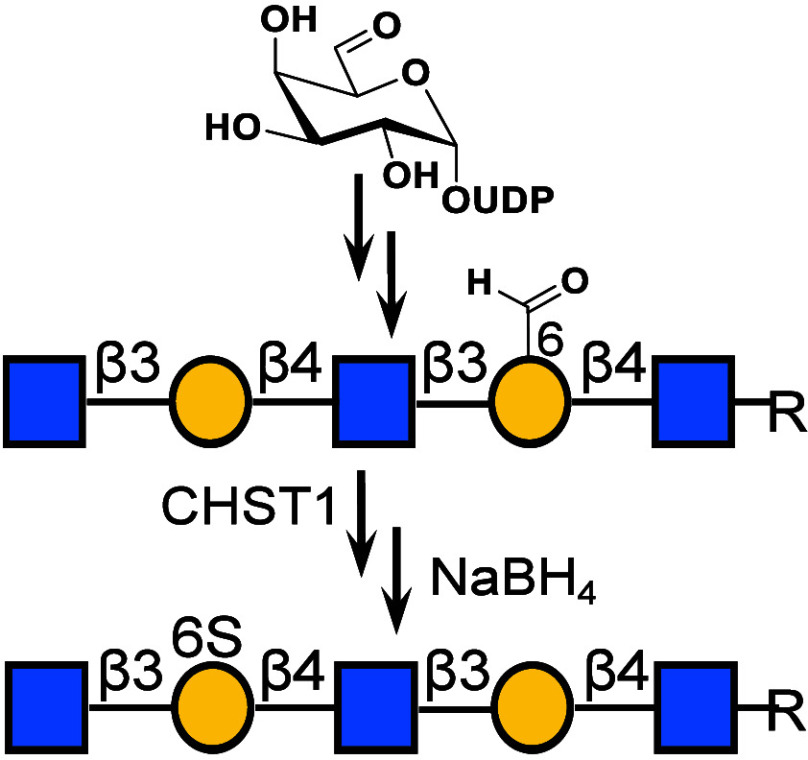

Keratan sulfate (KS) is a highly complex proteoglycan that has a poly-LacNAc chain that can be modified by diverse patterns of sulfate esters at C-6 positions of galactoside (Gal) and *N*-acetylglucosamine (GlcNAc) residues. Here, a chemo-enzymatic methodology is described that can control the pattern of sulfation at Gal using UDP-Gal-aldehyde as a donor for poly-LacNAc assembly to temporarily block specific sites from sulfation by galactose 6-sulfotransferase (CHST1).

Keratan sulfates (KS) are a family of proteoglycans that are decorated with polysaccharides composed of the repeating disaccharide [→3Galβ(1→4)GlcNAcβ(1→] (Figure 1A). C-6 hydroxyl of *N*-acetyl glucosamine (GlcNAc) and galactoside (Gal) moieties of these polysaccharides can be modified by a sulfate, and furthermore, GlcNAc can also bear an additional α1,3-fucoside.^1,2^ The termini of these polysaccharides can be capped by various epitopes, such as 2,3- and 2,6-sialosides.^3,4^ The KS biosynthetic enzymes cooperate to construct specific epitopes, and for example, GlcNAc-6-*O*-sulfotransferase 2 (CHST2) only sulfates terminal GlcNAc moieties to give GlcNAc6S that can be galactosylated by B4GalT4 to form βGal(1,4)GlcNAc6S. Furthermore, galactose 6-sulfotransferase (KSGal6ST, CHST1) can modify internal galactosides; however, its reactivity is much greater for galactosides neighbored by GlcNAc6S, and in particular, a Gal moiety flanked by GlcNAc6S and 2,3-Neu5Ac is preferentially sulfated.^5,6^

**Figure 1:**
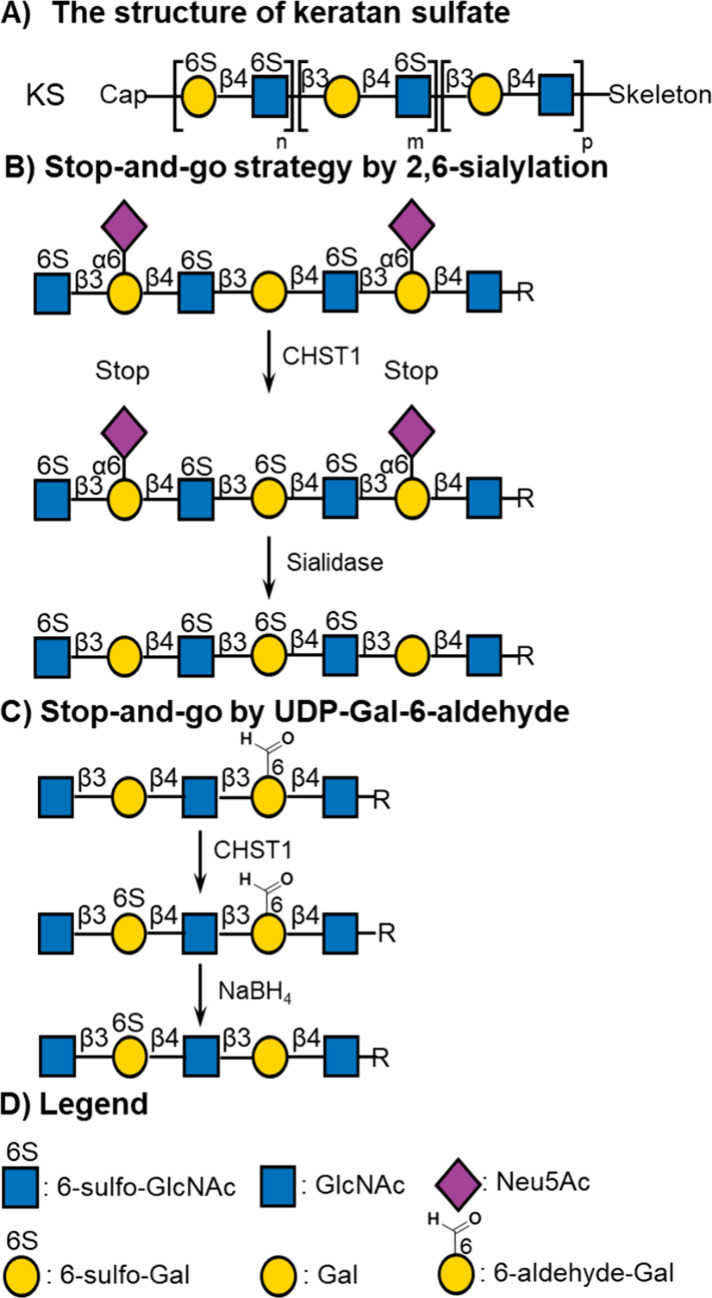
KS structure and chemoenzymatic synthesis strategy. (a) KS are *N*- and *O*-glycans having a sulfated poly-LacNAc chain. (b) Previously developed stop-and-go strategy for selective sulfation of Gal by 2,6-sialylation. (c) New stop-and-go strategy for selective sulfation of Gal using UDP-Gal-6-aldehyde. (d) Legend of monosaccharide structures.

KS is highly abundant in tensional and weight-bearing connective tissues (cornea, bone, cartilage, intervertebral disc, and tendon) and also expressed by epithelial cells and the central and peripheral nervous systems (CNS/PNS).^7^ It plays a role in many biological processes,^2,3,8,9^ for example, as a hydrating and signaling component in cornea and cartilage tissues.^2,9,10^ It regulates inflammation and is associated with rheumatoid arthritis, asthma, and chronic obstructive pulmonary disease.^11,12^ It is also involved in neurodegenerative diseases,^13^ such as amyotrophic lateral sclerosis (ALS) and Alzheimer’s disease.^14,15^

Owing to its structural complexity and its involvement in a myriad of biological functions, the synthesis of well-defined KS oligosaccharides has received considerable attention. Full chemical approaches have yielded several sulfated di- and tetrasaccharides.^16−20^ Furthermore, chemo-enzymatic methods have been described, in which chemically synthesized sulfated LacNAc derivatives were enzymatically fucosylated and sialylated to provide several KS epitopes.^21^ More complex structures have been obtained by chemical synthesis of oxazolines that could be transferred to an acceptor by a mutant keratanase II.^22,23^ Also, polyLacNAc chains have been enzymatically assembled followed by chemical sulfation of C-6 hydroxyls.^24^

Recently, we described a biomimetic approach that made it possible, for the first time, to prepare a large panel of highly complex oligo-*N*-acetyllactosamine (LacNAc) chains and *N*-glycans derived from KS having different patterns of sulfation and fucosylation.^25^ It exploits the substrate specificity of recombinant sulfotransferase CHST2, which modifies only terminal GlcNAc moieties to give GlcNAc-6S that could be galactosylated by B4Gal4 to give 6-sulfated LacNAc. Furthermore, it makes use of the observation that α1,3-fucosides, α2,6-sialosides, and C-6 sulfation of galactose (Gal6S) are mutually exclusive and cannot occur on the same LacNAc moiety. We demonstrated that, using specific enzyme modules, the various substructures observed in KS can assemble in any possible order and then be capped by the different terminal epitopes.

A key strategic principle of the biomimetic approach is the use of bacterial α2,6-sialyltransferase from *Photobacterium damselae* (Pd2,6ST) that can sialylate terminal as well as internal galactosides.^26,27^ It made it possible to temporarily block specific sites from sulfation by CHST1 (Figure 1B). At an appropriate stage of the synthesis, the sialosides could be removed by promiscuous neuraminidase from *Clostridium perfringens* and various terminal epitopes introduced, such as α2,3-sialoside or α2,6-sialoside using human recombinant sialyltransferases.

Here, we explore an alternative approach to block specific sites of an oligo-LacNAc chain from sulfation by CHST1 (Figure 1C). It is based on the use of unnatural UDP-Gal-6-aldehyde (**2**), which can be transferred by the bacterial galactosyltransferase from *Helicobacter pylori* β4GalT (Hpβ4GalT).^28^ The resulting terminal Gal-aldehyde is an appropriate substrate for β3GlcNAcT from *H. pylori* (Hpβ3GlcNAcT) to install a β1,3-linked GlcNAc moiety. The findings made it possible to assemble oligo-LacNAc chains having different patterns of natural and oxidized Gal moieties by employing UDP-Gal or UDP-Gal-6-aldehyde. As anticipated, sulfotransferase CHST1 in the presence of 3′-phosphoadenosine-5′-phosphosulfate (PAPS) modified only the natural Gal moieties. The aldehydes of the resulting sulfated oligosaccharides can be reduced to alcohols to give compounds with different patterns of sulfation. The approach streamlines the masking of specific galactosides from sulfation by CHST1.

Galactose oxidase (GAOX) is a fungal enzyme belonging to the family of oxidoreductases that can oxidase galactose to the corresponding aldehyde.^29^ The reaction is coupled with the reduction of oxygen to give hydrogen peroxide, which by fungi is employed as a bacteriostatic agent. GAOX is an attractive tool for chemoenzymatic synthesis and can selectively oxidize the terminal galactosides of poly-LacNAc chains. In an attractive application, galactose-6-aldehyde containing oligosaccharides was assembled by oxidation of terminal Gal of a LacNAc chain to the corresponding Gal-aldehyde that could be labeled with biotin–hydrazide. The aldehydes were also exploited for cross-linking with an amino-functionalized poly-LacNAc oligomer by reductive amination.^30−33^ GAOX has also been employed in redox-controlled enzymatic glycosylations. In this approach, C-6 hydroxyl of a non-reducing terminal Gal residue is oxidized to an aldehyde (Gal-6-aldehyde) followed by further assembly of a LacNAc chain having natural Gal and Gal-6-aldehyde residues. The resulting aldehydes allowed site-specific enzymatic α2,6-sialylation and α1,3-fucosylation of natural galactosides.^34,35^ Oxidation of C-6 hydroxyl of a terminal galactoside is, however, demanding, and there is a risk of side product formation, such elimination and over oxidation, to a carboxylic acid.^29,30^ The C-6 hydroxymethyl group of UDP-galactose has also been oxidized by galactose oxidase to the corresponding aldehyde and immediately employed in a subsequent reaction for different purposes.^36−39^

Many glycosyltransferases exhibit substrate promiscuities and can transfer sugar nucleotides having an unnatural modification.^40,41^ Such promiscuities have been exploited in the so-called chemical reporter strategy for labeling glycoconjugates in living cells with abiotic functionalities, such as an azide.^42^ We have introduced a chemoenzymatic strategy that was coined “stop-and-go chemoenzymatic glycosylation”, in which unnatural sugar nucleotide donors are employed in glycosyltransferase-mediated glycosylations to give a product in which the newly introduced unnatural monosaccharide is temporarily disabled (stop) from further enzymatic modification. At an appropriate stage of the synthesis, the unnatural monosaccharide can be converted into the natural counterparts (“go”) for further modification. By careful synthetic planning, the stop-and-go approach makes it possible to prepare highly complex oligosaccharides.^43−46^

On the basis of these advances, we were compelled to explore whether UDP-Gal-aldehyde (**2**) can be obtained by oxidation of UDP-Gal (**1**) by GAOX and then employed in the assembly of oligo-LacNAc derivatives. It was anticipated that the aldehydes would block specific sites from sulfation by CHST1. In this approach, only one challenging purification must be performed, and the resulting common UDP-Gal-aldehyde can be used for the preparation of various Gal-aldehyde-containing compounds.

Thus, UDP-Gal (**1**) was incubated with GOAX in the presence of peroxidase of horseradish peroxidase for 48 h, resulting in full consumption of compound **1**, to give, after purification by P2 size-exclusion column chromatography followed by preparative high-performance liquid chromatography (HPLC) over a SeQuant ZIC HILIC column, homogeneous compound **2** as a hydrate in a yield of 57% (46 mg) (Scheme 1a). Next, different mammalian and bacterial glycosyltransferases were tested for their tolerance to utilize compound **2** as a donor (Scheme 1b).^28,47^ It was observed that *Neisseria meningitidis* β4GalT (NmLgtB) does not transfer UDP-Gal-6-aldehyde. Similarly, the human galactosyl transferase, β4GalT1, does not readily accept compound **2**, and only partial conversion into compound **4** was observed after a prolonged incubation time. Gratifyingly, Hpβ4GalT exhibited a rate of transfer comparable to that of natural UDP-Gal and made it possible to prepare compound **4** in a high yield. Compound **4** was purified by size-exclusion column chromatography over Bio-Gel P2 using Mill-Q water as a solvent. It could be elongated by β3GlcNAcT from *H. pylori* (Hpβ3GlcNAcT) in the presence of UDP-GlcNAc to give trisaccharide **5**.^25,28^

**Scheme 1:**
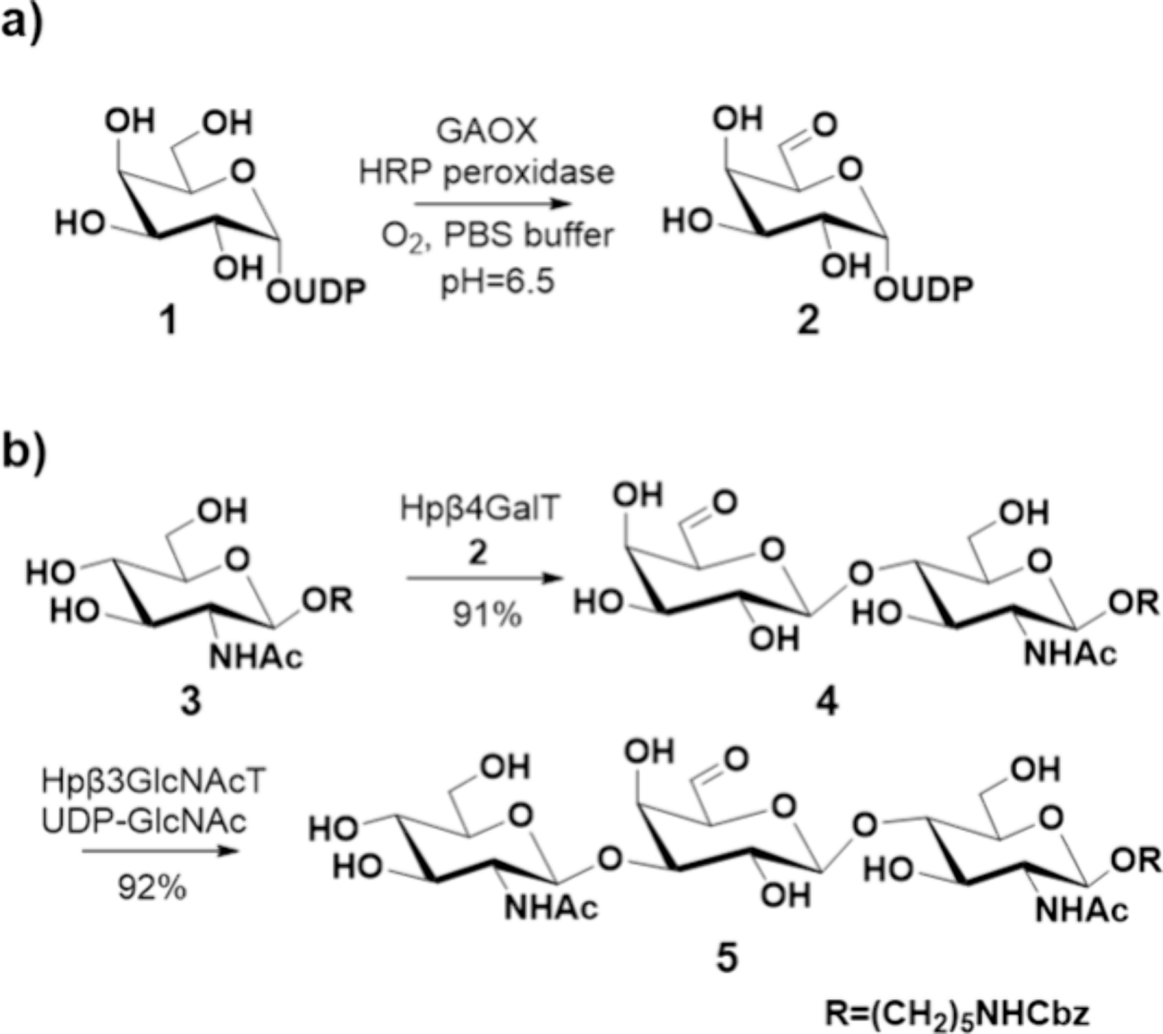
Synthesis of UDP-Gal-6-aldehyde **2** and Acceptor Properties

Another cycle of enzymatic modification by Hpβ4GalT (→**6**) and Hpβ3GlcNAcT gave pentasaccharide **7**. The two Gal-6-aldehyde caging moieties of compound **7** could readily be reduced to hydroxyls by aqueous NaBH_4_ to give natural backbone glycan **8**. The reaction mixture was slowly neutralized, followed by size-exclusion column chromatography, to afford compound **8** in 98% yield. Detailed nuclear magnetic resonance (NMR) analysis confirmed the structural integrity of the compounds. For example, a combination of ^1^H, correlation spectroscopy (COSY), nuclear Overhauser effect spectroscopy (NOESY), total correlation spectroscopy (TOCSY), and heteronuclear single-quantum correlation (HSQC) NMR experiments for compound **6** made it possible to assign all proton and carbon signals. It indicated that the aldehydes occur as a hydrate by comparing to spectra of the corresponding galactoside.^6,25^ C6 carbon of internal galactose-6-hydrate of the B moiety had substantially shifted downfield (δ 61.1 → δ 87.8), and the corresponding protons also exhibited a chemical shift difference (H6a and H6b 3.76 → H6 5.14). Nearby Gal B H-5 shifted from δ 3.72 to δ 3.45, which is indicative of the involvement of this proton in the nearby hydrate. Nearby Gal B H-4 also shifts from δ 4.16 to δ 4.32, which indicates the involvement of this proton in the nearby hydrate. The 6-carbon of the terminal galactose hydrate of the D moiety had substantially shifted downfield (δ 61.1 → δ 88.1), and the corresponding protons also exhibited a chemical shift difference (H6a and H6b 3.76 → H6 5.14). Nearby Gal D H-5 also shifts from δ 3.72 to δ 3.47, which indicates the involvement of this proton in the nearby hydrate. Nearby Gal D H-4 also shifts from δ 3.93 to δ 4.10.

Next, we explored whether oligosaccharides having a galactose-6-aldehyde moiety can be selectively sulfated by CHST1 (Scheme 2b). For this purpose, compound **9** was prepared by galactosylation of terminal GlcNAc of compound **8** by Hpβ4GalT and UDP-Gal-6-aldehyde (**2**), which reacted with CHST1 and PAPS to give disulfated compound **10**. The aldehyde moiety of the latter compound was reduced to Gal by treatment with aqueous NaBH_4_ to provide disulfated hexasaccharide **11** in a high yield. The same “stop-and-go” controlling modular strategy was successfully applied for the synthesis of monosulfated pentasaccharide **15**. In this sequence of reactions, compound **5** was elongated to compound **12** by β4GalT1 followed by subsequent modification by B3GNT2 to provide compound **13**. As expected, the natural Gal moiety of compound **13** could be selectively sulfated by CHST1 in the presence of PAPS to give compound **14**, which was treated with aqueous NaBH_4_ to provide compound **15**. These results highlight that Gal-6-aldehyde can block sulfation without hindering to reactivity of the nearby natural Gal moieties (+1, −1, or −2 site). Intermediate **15** was employed in a formal synthesis of a KS-derived oligosaccharide by treatment with CHST2 in the presence of PAPS, resulting in the facile formation of compound **16** (Scheme 3). This reaction exploits the fact that CHST2 will only sulfate terminal GlcNAc moieties. Terminal GlcNAc of compound **16** was extended by subsequent modification by B4GalT4 in the presence of UDP-Gal to give compound **17**, which was further modified by ST6Gal1 and CMP-Neu5Ac to provide compound **18**.

**Scheme 2:**
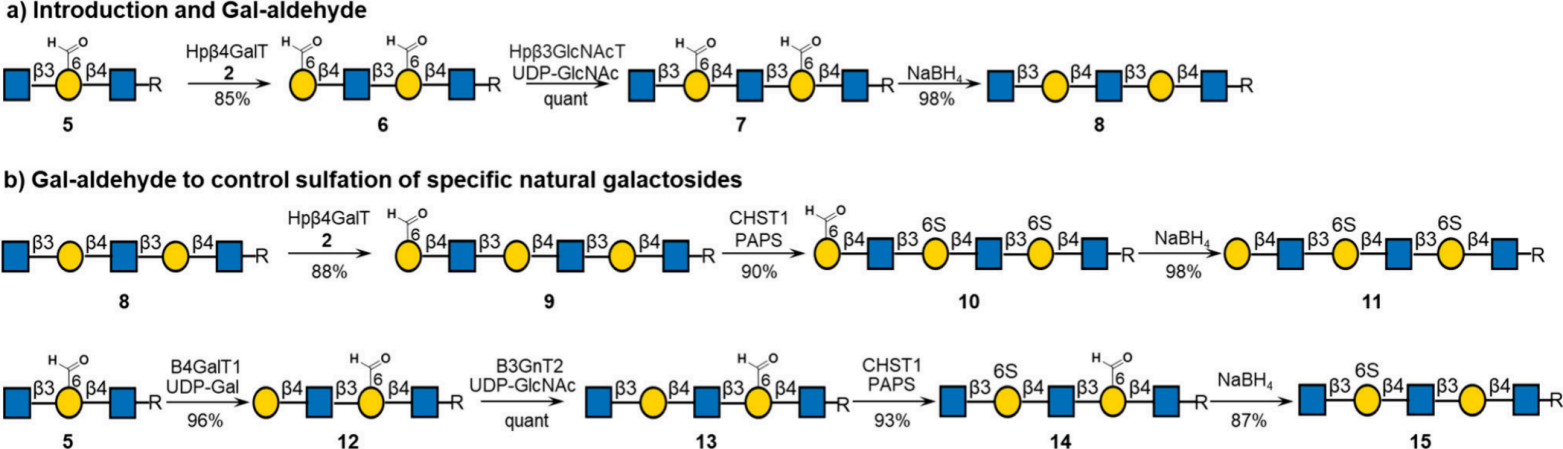
Enzymatic Synthesis of Selectively Sulfated KS Oligosaccharides [R = (CH_2_)_5_NHCbz]

**Scheme 3:**
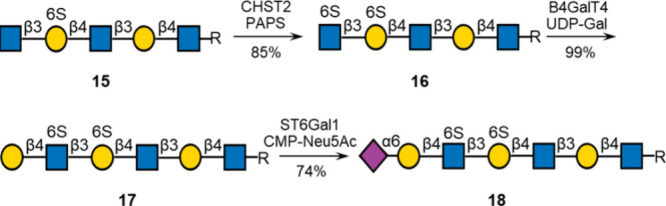
Formal Synthesis of KS-Derived Oligosaccharide **18**

The methodology described here makes it possible to prepare KS oligosaccharides having intricate patterns of sulfation by fewer steps and with improved atom economy compared to our previous reported biomimetic strategy.^25^ Careful manipulation of intermediates is, however, required to avoid β-elimination to form α,β-unsaturated aldehydes.^30,48^ It has been shown that Gal-6-aldehyde can be easily installed in oligosaccharides first by producing the unnatural sugar nucleotide, UDP-Gal-6-aldehyde, which can be employed as a donor substrate by galactosyl transferase, Hpβ4GalT. Highly complex KS oligosaccharides can easily be synthesized by exploiting Gal-6-aldehyde to control sites of sulfation as well as positions of fucosides.^34,35^

## Data Availability

The data underlying this study are available in the published article and its Supporting Information.

## Supporting Information Available

Complete experimental and spectral characterization [NMR and mass spectrometry (MS)] details and copies of ^1^H and ^1^H–^13^C HSQC NMR spectra of all new compounds (PDF)
