# Supplementary material for: Chemoenzymatic Synthesis of Keratan Sulfate Oligosaccharides Using UDP-Galactose-6-aldehyde To Control Sulfation at Galactosides
Source: Org Lett. 2024 Sep 23;26(39):8272–7. doi: 10.1021/acs.orglett.4c02899 (PMC11459510; doi:10.1021/acs.orglett.4c02899)
Supplement: Supplementary file 1 — ol4c02899_si_001.pdf [file ol4c02899_si_001.pdf]

Supporting Information

Chemoenzymatic Synthesis of Keratan Sulfate Oligosaccharides using UDP-Galactose-6-Aldehyde to Control Sulfation at Galactosides

Yunfei Wu, Gerlof P. Bosman, Gaël M. Vos, Elif Uslu, Digantkumar Chapla, Chin Huang, Kelley W. Moremen, and Geert-Jan Boons*

Table of Contents

page

1) Materials and Methods S3

2) Analytical Data S8

3) Experimental Procedures and Analysis S13

4) References S27

5) NMR Spectra S28

1) Materials and Methods

Glycosyltransferases Hpβ3GlcNAcT and Hpβ4GalT were expressed and purified according to published protocols. [1-4] Galactose Oxidase from Dactylium dendroides (CAS: 9028-79-9, G7400-10KU) and Peroxidase from horseradish (CAS: 9003-99-0) were obtained from Sigma-Aldrich. Reagents were purchased from Sigma-Aldrich. Uridine 5'-diphosphogalactose (UDP-Gal), uridine 5'-diphospho-N-acetyl-glucosamine (UDP-GlcNAc) and cytidine-5'-monophospho-N-acetylneuraminic acid (CMP-Neu5Ac) were obtained from Roche Diagnostics [UDP-Gal: Cat# 07703562103; UDP-GlcNAc: Cat# 06369855103; CMPNeu5Ac: Cat# 05974003103]. Adenosine 3′-Phosphate 5′-Phosphosulfate (PAPS) were obtained from Merck [Cat# 118410, Purity≥80% by HPLC]. Progress of the reactions was monitored by liquid chromatography mass spectrometry system (LCMS) from Shimadzu (system controller: SCL10A-VP; HPLC pumps: LC10AD-VP; injector: SIL10AD-VP) using a ZIC HILIC column (ZeQuant, PEEK coated guard HPLC column, 3.5 µm particle size, 20x 2.1 mm). The LC system was attached to a Bruker Daltonics micro TOF-Q mass spectrometer. Mass spectra were recorded on either on an Applied Biosystems SCIEX MALDI TOF/TOF 5800 mass spectrometer, a Shimadzu Biotech Axima-CFR MALDI-TOF, or a high resolution Shimadzu LCMS-IT-TOF mass spectrometer. Reaction mixtures were purified using a size exclusion Biogel (P2) or Biogel (P6) resins from BioRad in Econo glass columns (0.7 x 30 cm / 1.5 x 30 cm / 1.5 x 50 cm/ 1.5 x 120 cm) coupled to a BioFrac fraction collector (BioRad). Carbohydrate-containing fractions were detected by thin layer chromatography and an appropriate staining reagent (15 mL AcOH and 3.5 mL p-Anisaldehyde in 350 mL EtOH and 50 mL H_2SO_4). If needed, further purification was performed by HPLC-MS using a ZIC HILIC column.

Expression and purification of recombinant human glycosyltransferases and sulfotransferases

Expression constructs were generated encoding the truncated catalytic domains of human glycosyltransferases (B4GALT1, B4GALT4, B3GNT2 and ST6GAL1) and sulfotransferases (CHST1 and CHST2) as NH_2-terminal fusion proteins in the pGEn2 expression vector essentially as described in prior studies.[5-8] Briefly, the fusion protein coding regions were comprised of a 25-amino acid signal sequence, an His_8 tag, AviTag, the "superfolder" GFP coding region, the 7-amino acid recognition sequence of the tobacco etch virus (TEV) protease followed by the respective catalytic domain regions (for human CHST1 (Uniprot ID: O43916) and CHST2 (Uniprot ID: Q9Y4C5) catalytic domain region comprising of 388 and 454 amino acid residues, respectively). The recombinant human glycosyltransferases and sulfotransferases were expressed as a soluble secreted proteins by transient transfection of suspension culture HEK293-F cells (FreeStyle™ 293-F cells, Thermo Fisher Scientific, Waltham MA) and purified by Ni^{2+}-NTA chromatography as previously described.[5-6] Each protein was concentrated to approximately 3 mg/mL using an ultrafiltration pressure cell (Millipore, Billerica, MA) with a 10-kDa molecular mass cutoff membrane. The enzymes were further purified by gel filtration on a Superdex G-75 column (GE Healthcare) preconditioned with a buffer containing 20 mM HEPES, 150 mM NaCl, 0.05% sodium azide, pH 7.0. Peak fractions of recombinant human enzymes were pooled, respectively, concentrated at 1 mg/mL and buffer exchanged with 20 mM HEPES, 100 mM NaCl, 0.05% sodium azide, pH 7.0, 10% glycerol. The final protein preparations were aliquoted and stored at -80 °C until use.

General protocols for enzymatic reactions

General procedure of oxidation for UDP-Gal-6-aldehyde (2)

The substrate (UDP-gal **1**, 0.1 mmol) was dissolved in sodium phosphate buffer (50 mM, pH 6.5). Then, galactose oxidase (155 U) and peroxidase (3220 U) were added. The solution was stirred at 30 ºC under gentle mixing at 300 rpm in oxygen atmosphere. The progress of the reaction was monitored by MALDI-TOF MS or ESI-TOF MS, and if starting material remained after 24 h, another portion of galactose oxidase and peroxidase were added until no starting material could be detected. The reaction was terminated by adding the same volume of ice-cold ethanol and incubating at 4 ºC for 30 min. The mixture was then centrifuged and the precipitates were removed. The supernatant containing the product was concentrated, purified by BioGel P-2 column (eluted with H_2O) to provide purified product **2**. High performance liquid chromatography (HPLC) using a HILIC column (see materials) was employed for further purification.

General procedure for the installation of β1,3 GlcNAc using B3GnT2

Glycosyl acceptor (1.0 eq) and UDP-GlcNAc (1.5 eq) were dissolved to provide a final acceptor concentration of 2-5 mM in a HEPES buffer (50 mM, pH 7.0) containing KCl (25 mM), $MgCl_2$ (2 mM) and DTT (1 mM). Calf intestine alkaline phosphotase (CIAP, 1% total volume, 1 kU/mL) and B3GnT2 (1% wt/wt relative to acceptor substrate) were added, and the reaction mixture was incubated overnight at 37 °C with gentle shaking. The progress of the reaction was monitored by MALDI-TOF MS or ESI-TOF MS, and if starting material remained after 18 h, another portion of B3GnT2 was added until no starting material could be detected. The reaction mixture was centrifuged over a Nanosep® Omega ultrafiltration device (10 kDa MWCO) to remove proteins and the filtrate was lyophilized. The residue was applied to P2 or P6 size-exclusion column chromatography using Milli–Q water as eluent, providing the desired product. High performance liquid chromatography (HPLC) using a HILIC column (see materials) was employed when impurities were detected.

General procedure for the installation of β1,3 GlcNAc using Hpβ3GlcNAcT

Glycosyl acceptor (1.0 eq) and UDP-GlcNAc (1.5 eq) were dissolved to a final acceptor concentration of 2-5 mM in a HEPES buffer (50 mM, pH 7.0) containing KCl (25 mM), MgCl2 (2 mM) and DTT (1 mM). Calf intestine alkaline phosphotase (CIAP, 1% total volume, 1 kU/mL) and Hpβ3GlcNAcT (1% wt/wt relative to acceptor substrate) were added, and the reaction mixture was incubated overnight at 37 °C with gentle shaking. The progress of the reaction was monitored by MALDI-TOF MS or ESI-TOF MS, and if starting material remained after 18 h, another portion of Hpβ3GlcNAcT was added until no starting material could be detected. The reaction mixture was centrifuged over a Nanosep® Omega ultrafiltration device (10 kDa MWCO) to remove proteins and the filtrate was lyophilized. The residue was applied to P2 or P6 size-exclusion column chromatography using Milli–Q water as eluent, providing the desired product. High performance liquid chromatography (HPLC) using a HILIC column (see materials) was employed when impurities were detected.

General procedure for the installation of β1,4 Gal using B4GalT1

Glycosyl acceptor (1.0 eq) and UDP-Gal (1.5 eq) were dissolved to provide a final acceptor concentration of 2-5 mM in a Tris buffer buffer (100 mM, pH 7.0) containing $MnCl_2$ (10 mM) and BSA (1% total volume). CIAP (1% volume total) and B4GalT1 (1% wt/wt relative to acceptor substrate) were added, and the reaction mixture was incubated overnight at 37 °C with gentle shaking. The progress of the reaction was monitored by MALDI-TOF MS or ESI-TOF MS, and if starting material remained after 18 h, another portion of B4GalT1 was added until no starting material was detected. The reaction mixture was centrifuged over a Nanosep® Omega ultrafiltration device (10 kDa MWCO) to remove proteins and the filtrate was lyophilized. The residue was applied to P2 or P6 size-exclusion column chromatography using Milli–Q water as eluent, providing the desired product. High performance liquid chromatography (HPLC) using HILIC column (see materials) was employed when the impurities were founded after size exclusion.

General procedure for the installation of β1,4 Gal using B4GalT4

Glycosyl acceptor (1.0 eq) and UDP-Gal (1.5 eq) were dissolved to provide a final acceptor concentration of 2-5 mM in a Tris buffer buffer (100 mM, pH 7.0) containing $MnCl_2$ (10 mM) and BSA (1% total volume). CIAP (1% volume total) and B4GalT4 (1% wt/wt relative to acceptor substrate) were added, and the reaction mixture was incubated overnight at 37 °C with gentle shaking. The progress of the reaction was monitored by MALDI-TOF MS or ESI-TOF MS, and if starting material remained, another portion of B4GalT4 was added until no starting material could be detected. The reaction mixture was centrifuged over a Nanosep® Omega ultrafiltration device (10 kDa MWCO) to remove proteins and the filtrate was lyophilized. The residue was applied to P2 or P6 size-exclusion column chromatography using Milli–Q water as eluent, providing the desired product. High performance liquid chromatography (HPLC) using HILIC column (see materials) was employed when the impurities were detected after size exclusion column chromatography.

General procedure for the installation of β1,4 Gal-6-aldehyde using Hpβ4GalT

Glycosyl acceptor (1.0 eq) and UDP-galactose-6-aldehyde **2** (1.5 eq) were dissolved to provide a final acceptor concentration of 2-5 mM in a Tris buffer buffer (100 mM, pH 7.0) containing $MnCl_2$ (10 mM) and BSA (1% total volume). CIAP (1% volume total) and Hpβ4GalT (1% wt/wt relative to acceptor substrate) were added, and the reaction mixture was incubated overnight at 37 °C with gentle shaking. The progress of the reaction was monitored by MALDI-TOF MS or ESI-TOF MS, and if starting material remained, another portion of Hpβ4GalT was added until no starting material could be detected. The reaction mixture was centrifuged over a Nanosep® Omega ultrafiltration device (10 kDa MWCO) to remove proteins and the filtrate was lyophilized. The residue was applied to P2 or P6 size-exclusion column chromatography using Milli–Q water as eluent, providing the desired product. High performance liquid chromatography (HPLC) using HILIC column (see materials) was employed when the impurities were detected after size exclusion column chromatography.

General procedure for the installation of terminal α2,6 Neu5Ac using ST6Gal1

Glycosyl acceptor (1 eq) and CMP-Neu5Ac (1.5 eq) were dissolved in a Hepes buffer (100 mM, pH 7.5) containing BSA (1% volume total) to an acceptor concentration of 2 – 5 mM. CIAP (1% volume total) and ST6Gal1 (1% wt/wt relative to acceptor substrate) were added, and the reaction mixture was incubated overnight at 37 °C with gentle agitation. The reaction mixture was centrifuged over a Nanosep® Omega ultrafiltration device (10 kDa MWCO) to remove proteins, and the filtrate was lyophilized. The residue was applied to P2 or P6 size-exclusion column chromatography using NH_4HCO_3 buffer (50 mM) as eluent provided the desired product. High performance liquid chromatography (HPLC) using a HILIC column (see materials) was employed when the impurities were detected.

General procedure for the 6-O-sulfate installation of terminal GlcNAc using CHST2

Glycosyl acceptor (1.0 eq) and PAPS (1.6 eq) were dissolved at a final acceptor concentration of 2-5 mM in a Tris buffered solution (100 mM, pH 7.5) containing $MgCl_2$ (10 mM). CHST2 (10-20% wt/wt relative to acceptor substrate) was added, and the reaction mixture was incubated overnight at 37 °C with gentle shaking. The reaction mixture was centrifuged over a Nanosep® Omega ultrafiltration device (10 kDa MWCO) to remove proteins, and the filtrate was lyophilized. The residue was applied to P2 or P6 size-exclusion column chromatography using NH_4HCO_3 buffer (50 mM) as eluent, providing the desired product. High performance liquid chromatography (HPLC) using a HILIC column (see materials) or DEAE ion exchange column was employed when impurities were detected.

General procedure for the 6-O-sulfate installation of internal Galactose using CHST1

Glycosyl acceptor (1.0 eq) and PAPS (1.6 eq per galactose) were dissolved at a final acceptor concentration of 2–5 mM in a Tris buffered solution (100 mM, pH 7.0) containing $MgCl_2$ (10 mM). CHST1 (10% wt/wt relative to acceptor substrate) were added, and the reaction mixture was incubated overnight at 37 °C with gentle shaking. The reaction mixture was centrifuged over a Nanosep® Omega ultrafiltration device (10 kDa MWCO) to remove proteins, and the filtrate was lyophilized. The residue was applied to P2 or P6 size-exclusion column chromatography using Milli–Q water as eluent, providing the desired product. High performance liquid chromatography (HPLC) using a HILIC column (see materials) was employed when the impurities were detected.

General procedure of reduction reaction of Gal-6-aldehyde

To a solution of acceptor (1.0 equiv.) in water (1 mL), sodium borohydride ($NaBH_4$, 5 equiv.) pre-dissolved in water was added. The reaction was stirred at room temperature for 1 h. The progress of the reaction was monitored by ESI-TOF MS. The reaction was then slowly neutralized to pH = 8.0 with 1 M HCl, lyophilized. The residue was applicated to P2 or P6 size-exclusion column chromatography using NH_4HCO_3 buffer (50 mM) as eluent, providing the desired product.

General procedure for Cbz deprotection from the linker with $Pd(OH)_2$ reduction

Palladium hydroxide on carbon (Degussa type, 20%, 1.5 times the weight of starting material) was added to a solution of starting material in H_2O (0.1% AcOH as additive). The mixture was placed under an atmosphere of hydrogen until ESI-LC-MS indicated completion of the reaction. The mixture was filtered through a spin filter and the residue was washed with H_2O. The filtrate was lyophilized to give the final product. P6 size-exclusion column chromatography was used for purification using 50 mM ammonium bicarbonate as eluent. Fractions containing compound were lyophilized to give the desired product.

General protocols for HILIC-HPLC purification

HILIC-HPLC purification conditions for compounds:

Semi-preparative HILIC-HPLC was applied on a Shimadzu (LC-20AT, SIL-20A, CBM-20A, SPD-20A, FRC-10A) LC-ESI-IT-TOF with a XBridge HILIC column, 5 µm, 10 x 250 mm at a flow rate of 3.6 mL/min, injection volume of 100 µL (10-20 mg/mL), with 0.2% of the flow is diverted to the ESI-MS detector using a splitter. The purification was performed using 5% Milli–Q water in MeCN (buffer B) and MeCN in 80% Milli–Q water (buffer A).

General conditions for a linear gradient were used as the eluent:

For UDP-galactose-6-aldehyde **2**

Time (min)	A (%)	B (%)
0	10	90
120	50	50

Semi-preparative HILIC-HPLC was applied on a Shimadzu (LC-20AT, SIL-20A, CBM-20A, SPD-20A, FRC-10A) LC-ESI-IT-TOF with a XBridge HILIC column, 5 µm, 10 x 250 mm at a flow rate of 3.6 mL/min, injection volume of 100 µL (10-20 mg/mL), with 0.2% of the flow is diverted to the ESI-MS detector using a splitter. The purification was performed using 10% 10 mM NH_4HCO_3 in MeCN (buffer B) and MeCN in 80% 10 mM NH_4HCO_3 (buffer A).

General conditions for a linear gradient were used as the eluent:

For linear glycans

Time (min)	A (%)	B (%)
0	10	90
90	50	50

2) Analytical Data

NMR nomenclature

NMR data was obtained at room temperature on a 600 MHz instrument from Bruker. The chemical shift δ is given in parts per million (ppm) and refers to tetramethyl silane and the residual solvent peak [^{1}H-NMR: δ(D_2O) =4.79 ppm]. NMR data is given as follows: ^{1}H-NMR: chemical shift (multiplicity, coupling constants, relative integral, functional group); ^{13}C data are extracted from HSQC spectra and given as follows: chemical shift. Multiplicity is defined as follows: s = singlet; d = doublet; t = triplet; m= multiplet. Signals were assigned by numbering the monosaccharide units starting at the reducing end of the oligosaccharide. The assignment was performed by using 2D-NMR spectra (COSY, HSQC, TOCSY, NOESY). The yield/concentration of the final products was determined by NMR spectroscopy using n-propanol as an internal standard. High resolution masses were measured on an Agilent 6560 Ion Mobility Q-TOF LC-MS system.

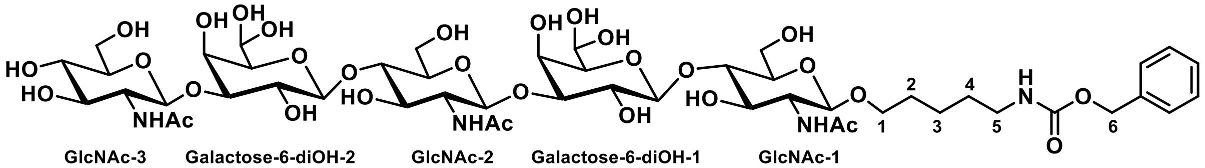

and

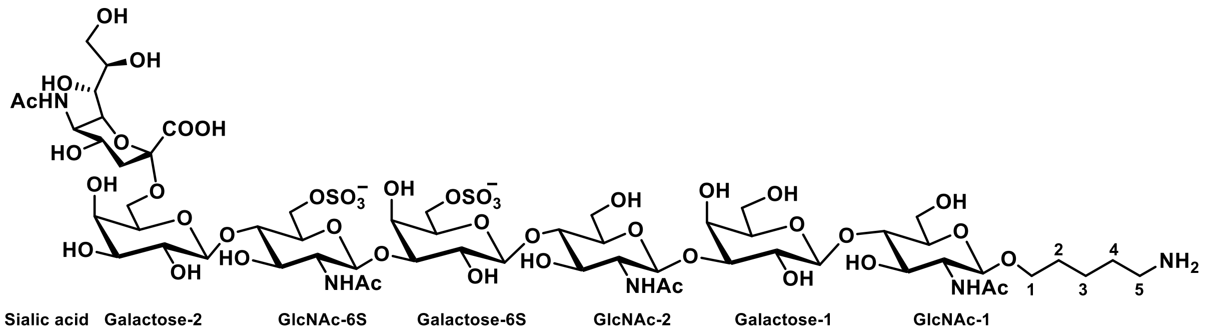

Labelling of linear glycans for NMR peak assignment. **A**, **B**, **C** and **D** labeling is also employed for tetrasaccharides. **A** refers to the monosaccharide at the reducing center followed by subsequent letter labeling.

Characterization of internal Gal-6-CH(OH)$_2$ of compound 5

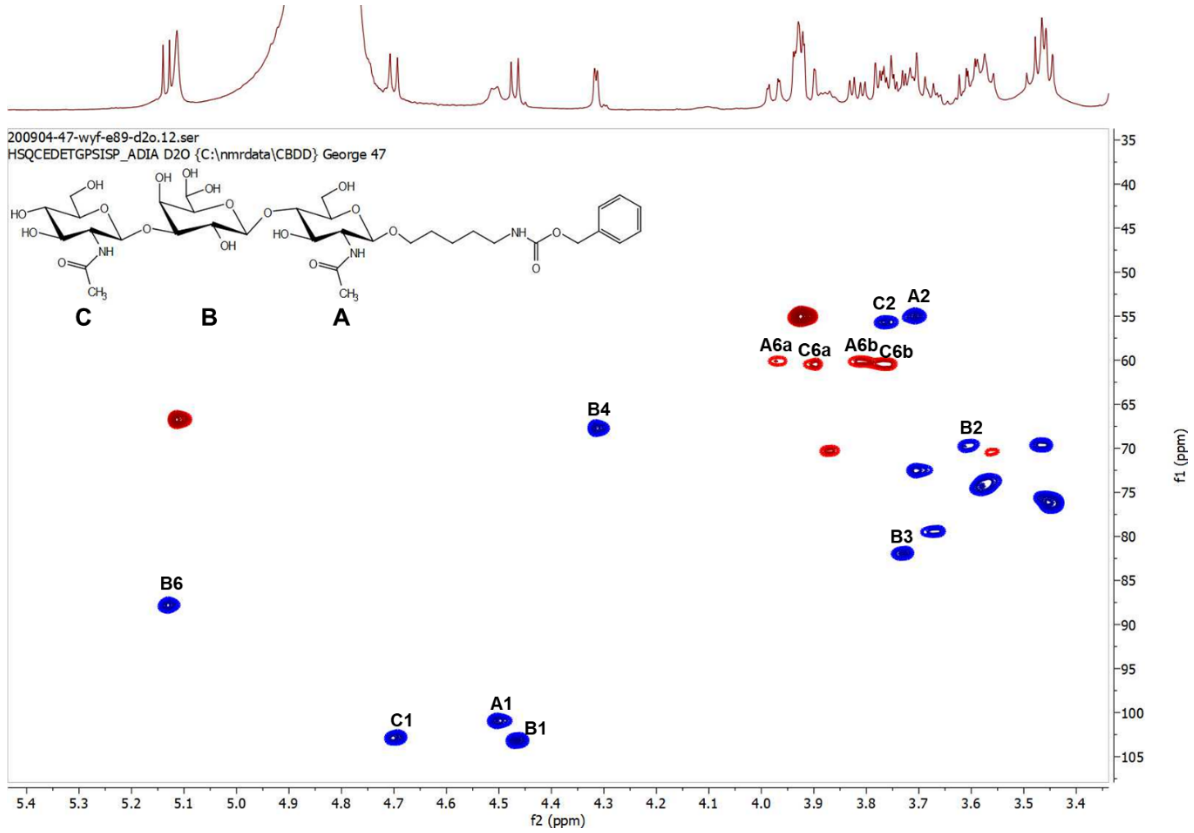

Figure S1a. 600 MHz 1D ^{1}H NMR and 2D ^{13}C-^{1}H HSQC spectra of **5**, recorded at 298K in D_2O.

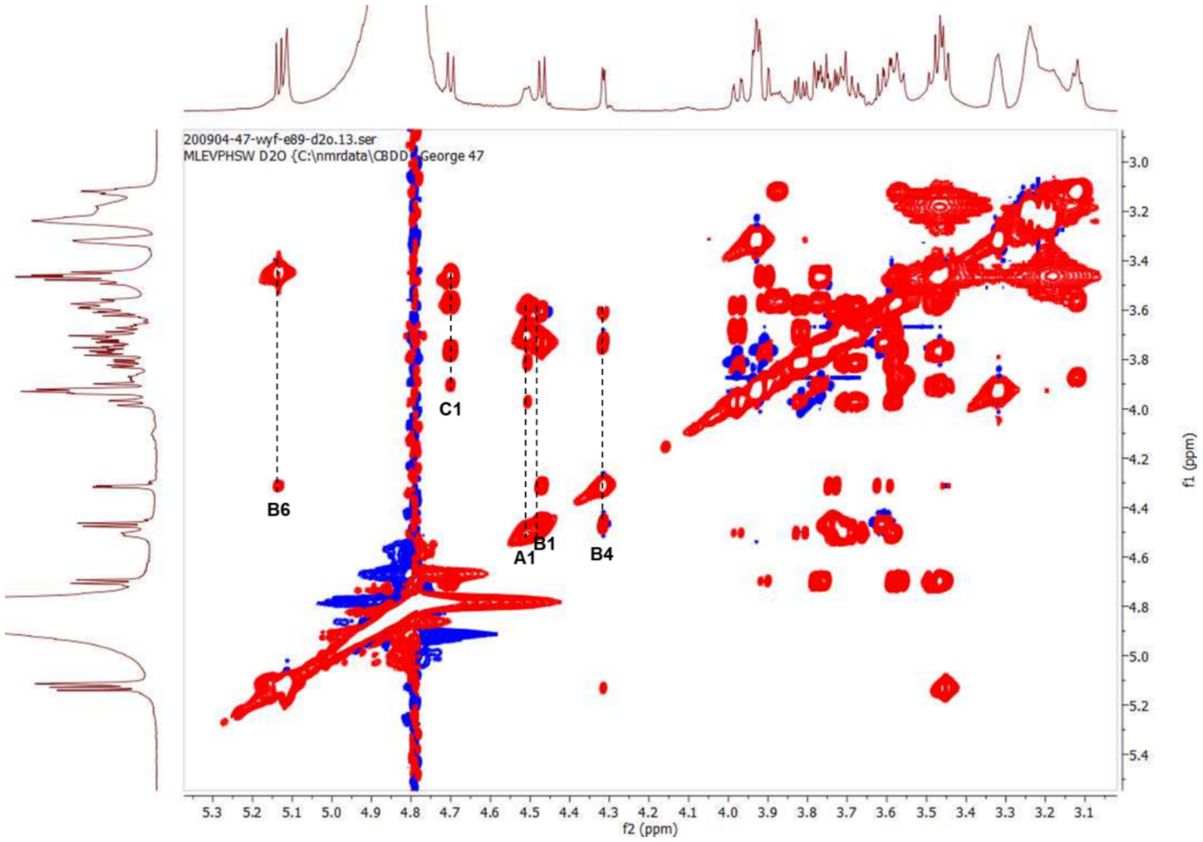

Figure S1b. 2D TOCSY (80 ms) spectrum of **5**.

The 1D ^{1}H NMR and 2D ^{13}C–^{1}H HSQC spectra of **5** are depicted in Figure S1a. The 2D TOCSY (80 ms) spectra of **5** is depicted in Figure S1b.

Detailed NMR analysis confirmed the GlcNAc and Galactose-6-diOH position of compound **5**. For example, 1D ^{1}H NMR and 2D ^{13}C–^{1}H HSQC spectra of compound **5** made it possible to assign all proton and carbon signals. The 1D ^{1}H NMR spectrum of **5** shows three anomeric signals, correlating to residues **A**, **B** and **C**. The H-1 signals at δH 4.51 (**A**) stems from a reducing-end GlcNAc residue. whereas the H-1 signal at δH 4.47 (**B**) belongs to non-reducing βGal. The anomeric signals at δH 4.70 (**C**) belongs to non-reducing βGlcNAc, respectively.

The position of the Galactose-6-diOH was confirmed by a combination of ^{1}H, COSY, NOESY, TOCSY and HSQC NMR experiments on compound **5**. Compared to reported corresponding galactose, the 6-carbon of Galactose-6-diOH the –CH_2– type group has been changed to –CH–. The 6-carbon of the internal Galactose-6-diOH moiety had substantially shifted downfield (δ 60.8 → δ 87.9) and the corresponding protons also exhibited a chemical shift difference (H6a and H6b 3.76 → H6 5.13). The nearby Gal H-5 also shifts from (H5 δ 3.72) to (H5 δ 3.46), which indicates the involvement of this proton in nearby diOH group. The nearby Gal H-4 also shifts from (H4 δ 4.16) to (H4 δ 4.31), which indicating the involvement of this proton in nearby diOH group.

The inter-residue connectivity was confirmed by a NOESY spectrum, the inter-residue connectivities GlcNAc-C H-1, Gal-**B** H-3 and Gal-**B** H-1, GlcNAc-**A** H-4 are in accordance with **C**(1→3)B, **B**(1→4)**A** linkages, respectively.

Characterization of internal and terminal Gal-6-CH(OH)$_2$ of compound 6

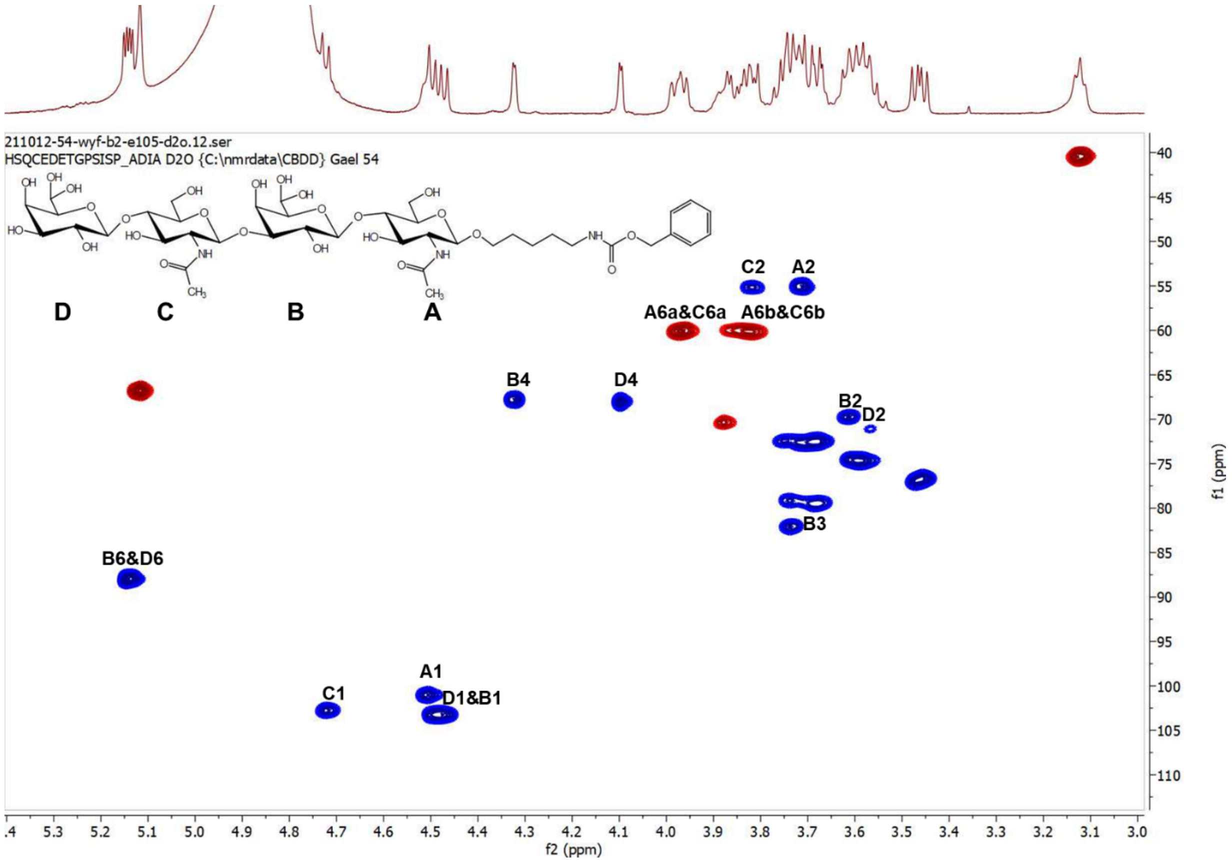

Figure S2a. 600 MHz 1D ^{1}H NMR and 2D ^{13}C-^{1}H HSQC spectra of **6**, recorded at 298K in D_2O.

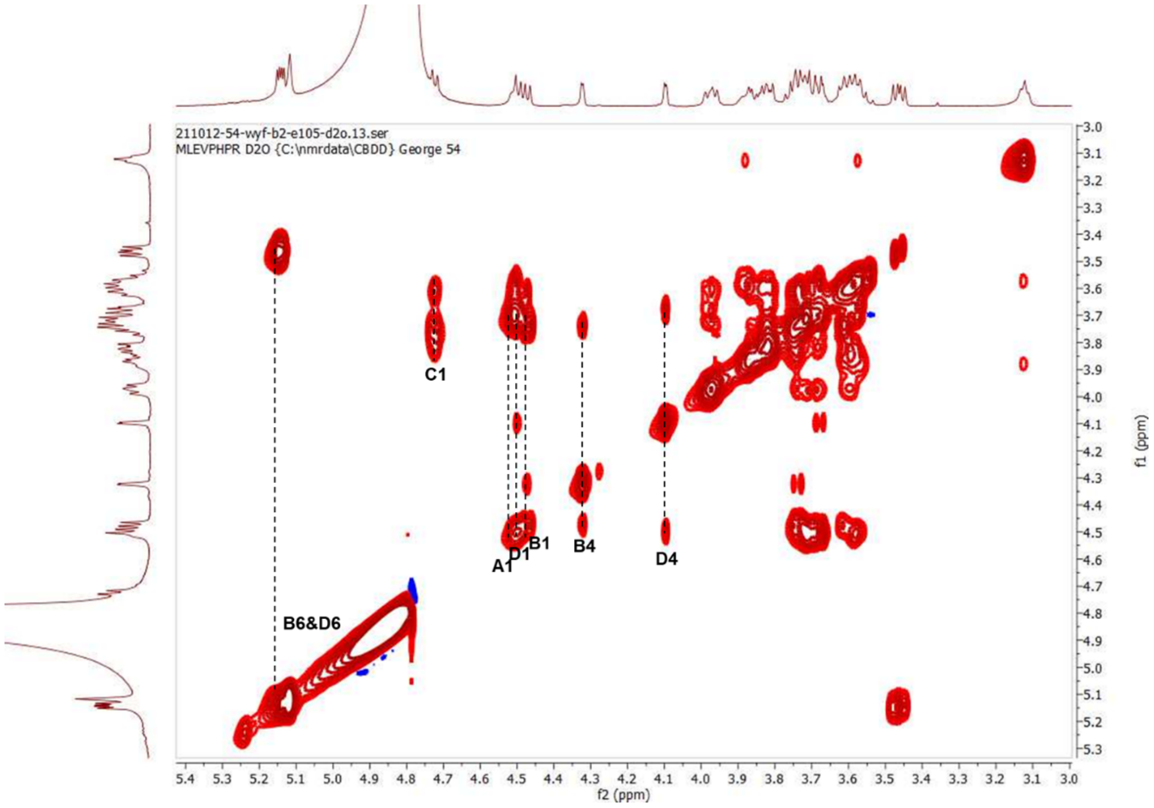

Figure S2b. 2D TOCSY (80 ms) spectrum of **6**.

The 1D ^{1}H NMR and 2D ^{13}C–^{1}H HSQC spectra of **6** are depicted in Figure S2a. The 2D TOCSY (80 ms) spectra of **6** is depicted in Figure S2b.

Detailed NMR analysis confirmed the GlcNAc and Galactose-6-diOH position of compound **6**. For example, 1D ^{1}H NMR and 2D ^{13}C–^{1}H HSQC spectra of compound **6** made it possible to assign all proton and carbon signals. The 1D ^{1}H NMR spectrum of **6** shows four anomeric signals, correlating to residues **A**, **B**, **C** and **D**. The H-1 signals at δH 4.51 (**A**) stems from a reducing-end GlcNAc residue. Whereas the H-1 signal at δH 4.47 (**B**) and the H-1 signal at δH 4.50 (**D**) belong to non-reducing βGal. The anomeric signals at δH 4.72 (b) belongs to non-reducing βGlcNAc.

The position of the Galactose-6-CH(OH)$_2$ was confirmed by a combination of ^{1}H, COSY, NOESY, TOCSY and HSQC NMR experiments on compound **6**. Compared to corresponding galactose, the 6-carbon of galactose-6-CH(OH)$_2$ has changed from –CH$_2$–to –CH–. The 6-carbon of the internal Galactose-6-diOH of the **B** moiety has substantially shifted downfield (δ 61.1 → δ 87.8) and the corresponding protons also exhibited a chemical shift difference (H6a and H6b 3.76 → H6 5.14). The nearby Gal **B** H-5 also shifts from (H5 δ 3.72) to (H5 δ 3.45), which indicating the involvement of this proton in nearby diOH group. The nearby Gal **B** H-4 also shifts from (H4 δ 4.16) to (H4 δ 4.32), which indicating the involvement of this proton in nearby diOH group. The 6-carbon of the terminal Galactose-6-diOH **D** moiety had substantially shifted downfield (δ 61.1 → δ 88.1) and the corresponding protons also exhibited a chemical shift difference (H6a and H6b 3.76 → H6 5.14). The nearby Gal **D** H-5 also shifts from (H5 δ 3.72) to (H5 δ 3.47), which indicating the involvement of this proton in nearby diOH group. The nearby Gal **D** H-4 also shifts from (H4 δ 3.93) to (H4 δ 4.10), which indicating the involvement of this proton in nearby diOH group.

The inter-residue connectivity was confirmed by a NOESY spectrum, the inter-residue connectivities Gal-**D** H-3, Gal-**D** H-1, GlcNAc-**C** H-4, GlcNAc-**C** H-1, Gal-**B** H-3 and Gal-**B** H-1, GlcNAc-**A** H-4 are in accordance with **D**(1→4)**C**, **C**(1→3)**B**, **B**(1→4)**A** linkages, respectively.

3) Experimental Procedures and Analysis

UDP-Galactose-6-diOH (2)

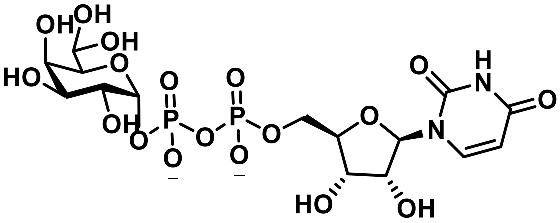

2 was prepared from **1** (79.1 mg, 0.14 mmol) using the general procedure of Oxidation for UDP-Gal-6-aldehyde 2. After P2 and HILIC HPLC purification, **2** was obtained as a white solid (46.8 mg, 57%). ^{1}H NMR (600 MHz, D_2O) δ 7.96 (d, J = 7.9 Hz, 1H, CO-CH=CH-N), 6.01 – 5.96 (m, 2H, H-1^{UDP}, CO-CH=CH-N), 5.66 (dd, J = 7.6, 3.6 Hz, 1H, H-1$^{Galactose\text{-}6diOH}$), 5.07 (d, J = 7.2 Hz, 1H, H-6$^{Galactose\text{-}6diOH}$), 4.39 – 4.36 (m, 2H, H-2^{UDP}, H-3^{UDP}), 4.29 (d, J = 2.9 Hz, 1H, H-4^{UDP}), 4.27 – 4.19 (m, 1H, H-5a^{UDP}, H-5b^{UDP}), 4.17 (d, J = 3.2 Hz, 1H, H-4$^{Galactose\text{-}6diOH}$), 3.93 – 3.91 (m, 1H, H-5$^{Galactose\text{-}6diOH}$), 3.92 – 3.89 (m, 1H, H-3$^{Galactose\text{-}6diOH}$), 3.82 (dt, J = 10.3, 3.1 Hz, 1H, H-2$^{Galactose\text{-}6diOH}$). ^{13}C NMR from HSQC (150 MHz, D_2O) δ 141.6 (CO-CH=CH-N), 102.7 (CO-CH=CH-N), 96.0 (C-1$^{Galactose\text{-}6diOH}$), 88.6 (C-6$^{Galactose\text{-}6diOH}$), 88.4 (C-1^{UDP}), 83.3 (C-4^{UDP}), 73.8 (C-2^{UDP}), 73.7 (C-5$^{Galactose\text{-}6diOH}$), 69.7 (C-3^{UDP}), 69.1 (C-3$^{Galactose\text{-}6diOH}$), 68.7 (C-4$^{Galactose\text{-}6diOH}$), 68.4 (C-2$^{Galactose\text{-}6diOH}$), 65.0 (C-5^{UDP}). ^{31}P NMR (162 MHz, D_2O) δ -11.24, -12.76. HRMS (ESI-MS): m/z calculated for $C_{15}H_{24}N_2O_{18}P_2$ [M-H]$^-$: 581.0426; found: 581.0439.

Compound 4

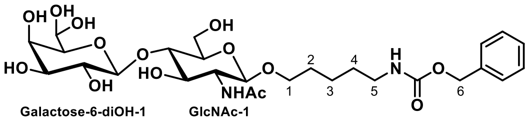

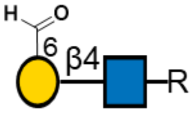

4 was prepared from **3** (3.0 mg, 6.8 μmol) using the general procedure for installation of β1,4 Gal-6-aldehyde using Hpβ4GalT. After P2 purification, **4** was obtained as a white solid (3.8 mg, 91%).

^{1}H (600 MHz, D_2O): δ (ppm)

	H-1	H-2	H-3	H-4	H-5	H-6	NHAc
GlcNAc	4.51 (d, J = 6.7 Hz, 1H)	3.72	3.70	3.70	3.58	3.98 (dd, J = 12.3, 2.3 Hz, 1H), 3.82 (dd, J = 12.2, 5.2 Hz, 1H)	2.00 (s, 3H)
Galactose-6-diOH	4.48 (d, J = 7.8 Hz, 1H)	3.57	3.69	4.09 (d, J = 3.3 Hz, 1H)	3.46 (d, J = 7.3 Hz, 1H)	5.13 (d, J = 7.4 Hz, 1H)	-

^{13}C (150 MHz, D_2O): δ (ppm)

	C-1	C-2	C-3	C-4	C-5	C-6	NHAc
GlcNAc	100.9	55.0	72.4	79.4	74.6	60.1	22.2

Galactose-6-diOH	103.2	71.0	72.4	68.0	76.9	88.0	-

Linker	1	2	3	4	5	6
H	3.88 (dt, J = 11.1, 5.9 Hz, 1H), 3.60 – 3.58 (m, 1H)	1.55 (p, J = 6.8 Hz, 2H)	1.32 (dt, J = 13.3, 6.1 Hz, 2H)	1.49 (p, J = 7.3 Hz, 2H)	3.12 (t, J = 6.8 Hz, 2H)	5.11 (s, 2H)
C	69.8	28.3	22.5	28.5	40.3	66.8

HRMS (ESI-MS): m/z calculated for $C_{27}H_{42}N_2O_{14}$ Na $[M+Na]^+$: 641.2529; found: 641.2530.

Compound 5

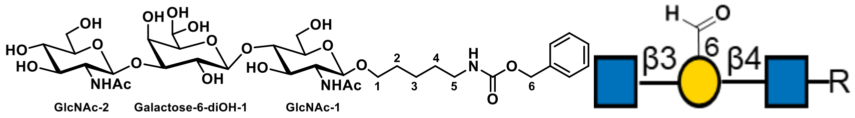

5 was prepared from **4** (8.3 mg, 13.4 μmol) using the general procedure for installation of β1,3 GlcNAc using Hpβ3GlcNAcT. After P2 purification, **5** was obtained as a white solid (10.2 mg, 92%).

^{1}H (600 MHz, D_2O): δ (ppm)

	H-1	H-2	H-3	H-4	H-5	H-6	NHAc
GlcNAc-1	4.51 (d, J = 6.2 Hz, 1H)	3.71	3.70	3.68	3.58	3.98, 3.82	2.00 (s, 3H)
Galactose-6-diOH	4.47 (d, J = 8.0 Hz, 1H)	3.61	3.74	4.31 (dd, J = 3.2 Hz, 1H)	3.46	5.13 (d, J = 7.3 Hz, 1H)	-
GlcNAc-2	4.70 (d, J = 8.4 Hz, 1H)	3.77	n/a	3.58	3.46	3.90, 3.76	2.05 (s, 3H)

^{13}C (150 MHz, D_2O): δ (ppm)

	C-1	C-2	C-3	C-4	C-5	C-6	NHAc
GlcNAc-1	101.1	55.1	72.6	79.5	74.6	60.4	22.1
Galactose-6-diOH	103.4	69.8	82.2	67.8	76.5	87.9	-
GlcNAc-2	102.9	55.8	n/a	n/a	n/a	60.6	22.1

Linker	1	2	3	4	5	6
H	3.87,3.55	1.59 – 1.52 (m, 2H)	1.35 – 1.28 (m, 2H)	1.49 (p, J = 7.3 Hz, 2H)	3.14 – 3.09 (m, 2H)	5.11 (s, 2H)
C	70.4	28.4	22.5	28.5	40.4	66.8

HRMS (ESI-MS): m/z calculated for $C_{35}H_{55}N_3O_{19}Na$ $[M+Na]^+$: 844.3322; found: 844.3374.

Compound 6

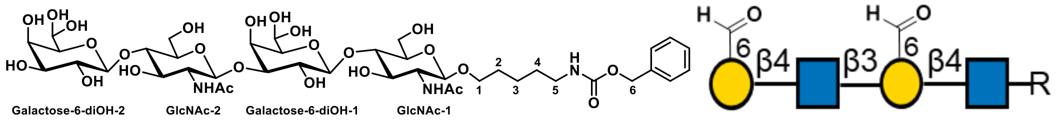

6 was prepared from **5** (2.5 mg, 3.0 μmol) using the general procedure for installation of β1,4 Gal-6-aldehyde using Hpβ4GalT. After P2 purification, **6** was obtained as a white solid (2.6 mg, 85%).

^{1}H (600 MHz, D_2O): δ (ppm)

	H-1	H-2	H-3	H-4	H-5	H-6	NHAc
GlcNAc-1	4.51	3.72	3.68	3.68	3.59	3.98, 3.83	2.01 (s, 3H)
Galactose-6-diOH-1	4.47 (d, J = 7.9 Hz, 1H)	3.62	3.72	4.32 (d, J = 3.2 Hz, 1H)	3.45 (d, J = 7.4 Hz, 1H)	5.14 (d, J = 7.5 Hz, 1H)	-
GlcNAc-2	4.72 (d, J = 8.3 Hz, 1H)	3.82	3.74	3.74	3.61	3.96, 3.85	2.04 – 2.03 (m, 3H)
Galactose-6-diOH-2	4.50 (d, J = 7.9 Hz, 1H)	3.57	3.67	4.10 (d, J = 3.3 Hz, 1H)	3.47 (d, J = 7.5 Hz, 1H)	5.14 (d, J = 7.3 Hz, 1H)	-

^{13}C (150 MHz, D_2O): δ (ppm)

	C-1	C-2	C-3	C-4	C-5	C-6	NHAc
GlcNAc-1	101.0	55.1	72.6	79.5	74.7	60.2	22.2
Galactose-6-diOH-1	103.3	69.7	82.1	67.7	76.2	87.8	-
GlcNAc-2	102.8	55.2	72.5	79.2	74.7	60.2	22.2
Galactose-6-diOH-2	103.3	71.1	72.5	68.1	77.1	88.1	-

Linker	1	2	3	4	5	6
H	3.87,3.57	1.59 – 1.52 (m, 2H)	1.35 – 1.28 (m, 2H)	1.49 (p, J = 7.3 Hz, 2H)	3.12 (t, J = 6.8 Hz, 2H)	5.12 (s, 2H)
C	70.6	28.2	22.4	28.4	40.5	66.7

HRMS (ESI-MS): m/z calculated for $C_{41}H_{65}N_3O_{25}$ Na $[M+Na]^+$: 1022.3800; found: 1022.3870.

Compound 7

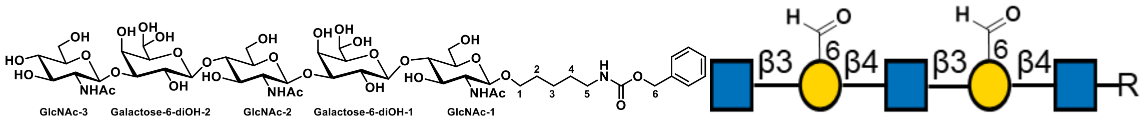

7 was prepared from **6** (2.3 mg, 2.3 μmol) using the general procedure for installation of β1,3 GlcNAc using Hpβ3GlcNAcT. After P2 purification, **7** was obtained as a white solid (2.8 mg, quant).

^{1}H (600 MHz, D_2O): δ (ppm)

	H-1	H-2	H-3	H-4	H-5	H-6	NHAc
GlcNAc-1	4.50 (d, J = 7.9 Hz, 1H)	3.71	3.68	3.68	3.58	3.97, 3.83	2.01 (s, 3H)
Galactose-6-diOH-1	4.46	3.61	3.74	4.31 (d, J = 3.2 Hz, 1H)	3.46	5.13 (d, J = 7.6 Hz, 1H)	-
GlcNAc-2	4.71	3.81	3.74	3.73	3.60	3.97, 3.83	2.06 – 2.03 (m, 6H)
Galactose-6-diOH-2	4.48	3.61	3.74	4.31 (d, J = 3.2 Hz, 1H)	3.46	5.14 (d, J = 7.3 Hz, 1H)	-
GlcNAc-3	4.70	3.77	n/a	3.57	3.46	3.91, 3.77	2.06 – 2.03 (m, 6H)

^{13}C (150 MHz, D_2O): δ (ppm)

	C-1	C-2	C-3	C-4	C-5	C-6	NHAc
GlcNAc-1	101.0	55.0	72.4	79.5	74.4	60.1	22.1
Galactose-6-diOH-1	103.3	69.7	82.1	67.8	76.4	87.8	-
GlcNAc-2	102.8	55.2	72.5	79.3	74.8	60.1	22.1
Galactose-6-diOH-2	103.3	69.7	82.1	67.8	76.4	88.0	-
GlcNAc-3	102.8	55.6	n/a	n/a	n/a	60.4	22.1

Linker	1	2	3	4	5	6
H	3.88,3.57	1.59 – 1.52 (m, 2H)	1.35 – 1.28 (m, 2H)	1.48 (p, J = 7.3 Hz, 2H)	3.12 (t, J = 6.5 Hz, 2H)	5.12 (s, 2H)
C	70.5	28.3	22.5	28.5	40.5	66.8

HRMS (ESI-MS): m/z calculated for $C_{49}H_{78}N_4O_{30}$ Na $[M+Na]^+$: 1225.4594; found: 1225.4698.

Compound 8

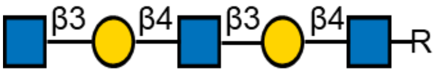

8 was prepared from **7** (0.8 mg, 0.7 μmol) using the general procedure of reduction reaction of Gal-6-aldehyde with sodium borohydride. After P2 purification, **8** was obtained as a white solid (0.8 mg, 98%). NMR data is confirmed with reference.

Compound 9

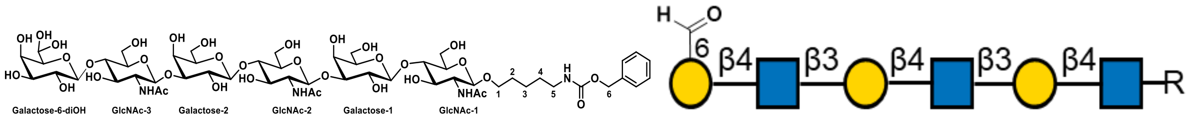

9 was prepared from **8** (0.8 mg, 3.0 µmol) using the general procedure for installation of β1,4 Gal-6-aldehyde using Hpβ4GalT. After P2 purification, **9** was obtained as a white solid (0.8 mg, 88%).

^{1}H (600 MHz, D_2O): δ (ppm)

	H-1	H-2	H-3	H-4	H-5	H-6	NHAc
GlcNAc-1	4.51	3.72	3.68	3.69	3.58	3.98, 3.82	2.00 (s, 3H)
Galactose-1	4.46	3.59	3.73	4.16	3.72	3.75 (4H)	-
GlcNAc-2	4.71	3.81	3.73	3.73	3.60	3.96, 3.84	2.05 – 2.03 (m, 6H)
Galactose-2	4.47	3.59	3.73	4.16	3.72	3.75 (4H)	-
GlcNAc-3	4.71	3.81	3.73	3.73	3.60	3.96, 3.84	2.05 – 2.03 (m, 6H)
Galactose-6-diOH	4.49	3.57	3.68	4.09 (dd, J = 3.3, 1.0 Hz, 1H)	3.47 (dd, J = 7.4, 1.1 Hz, 1H)	5.14 (d, J = 7.4 Hz, 1H)	-

^{13}C (150 MHz, D_2O): δ (ppm)

	C-1	C-2	C-3	C-4	C-5	C-6	NHAc
GlcNAc-1	101.0	55.0	72.4	78.4	74.8	59.9	22.1
Galactose-1	102.7	69.9	82.1	68.3	74.9	60.9	-
GlcNAc-2	102.7	55.1	72.2	78.6	74.6	59.9	22.1
Galactose-2	102.9	69.9	82.1	68.3	74.9	60.9	-
GlcNAc-3	102.7	55.1	72.1	78.6	74.6	59.9	22.1
Galactose-6-diOH	103.1	70.6	72.4	68.0	76.9	87.9	-

Linker	1	2	3	4	5	6
H	3.87, 3.55	1.59 – 1.52 (m, 2H)	1.34 – 1.28 (m, 2H)	1.49 (p, J = 7.3 Hz, 2H)	3.12 (t, J = 6.8 Hz, 1H)	5.11 (s, 2H)
C	70.3	28.4	22.4	28.5	40.3	66.8

HRMS (ESI-MS): m/z calculated for $C_{55}H_{88}N_4O_{34}Na$ $[M+Na]^+$: 1371.5173; found: 1371.5055.

Compound 10

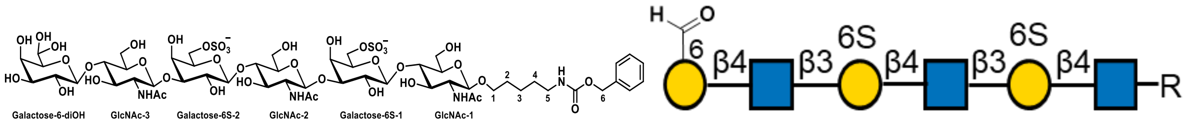

10 was prepared from **9** (0.4 mg, 0.3 μmol) using the general procedure for 6-O-sulfate installation of internal Galactose using CHST1. After P6 purification, **10** was obtained as a white solid (0.4 mg, 90%).

^{1}H (600 MHz, D$_2$O): δ (ppm)

	H-1	H-2	H-3	H-4	H-5	H-6	NHAc
GlcNAc-1	4.51	3.71	3.71	n/a	3.59	3.97, 3.83	2.00 (s, 3H)
Galactose-6S-1	4.51	3.59	3.74	4.23	3.97	4.22 – 4.18 (m, 4H)	-
GlcNAc-2	4.70	3.81	3.73	3.73	3.59	3.96, 3.86	2.04 (s, 6H)
Galactose—6S-2	4.51	3.59	3.74	4.23	3.97	4.22 – 4.18 (m, 4H)	-
GlcNAc-3	4.70	3.81	3.75	n/a	3.59	3.96, 3.86	2.04 (s, 6H)
Galactose-6diOH	4.49	3.56	3.70	4.09	3.47	5.14	-

^{13}C (150 MHz, D$_2$O): δ (ppm)

	C-1	C-2	C-3	C-4	C-5	C-6	NHAc
GlcNAc-1	101.1	55.3	72.5	n/a	74.9	60.3	22.2
Galactose-6S-1	103.3	70.1	82.6	68.3	72.7	67.4	-
GlcNAc-2	102.9	55.4	72.3	79.2	74.9	60.0	22.2
Galactose-6S-2	103.3	70.1	82.6	68.3	72.7	67.4	-
GlcNAc-3	102.9	55.4	72.3	n/a	74.9	60.0	22.2
Galactose-6diOH	103.2	n/a	n/a	68.0	77.0	88.1	-

Linker	1	2	3	4	5	6
H	3.87, 3.55	1.59 – 1.52 (m, 2H)	1.34 – 1.28 (m, 2H)	1.48 (p, J = 7.2 Hz, 2H)	3.16 – 3.08 (m, 2H)	5.12 (s, 2H)
C	n/a	28.3	n/a	28.5	40.5	66.7

HRMS (ESI-MS): m/z calculated for $C_{55}H_{86}N_4O_{40}S_2$ $[M-2H]^{2-}$: 753.2135; found: 753.2143.

Compound 11

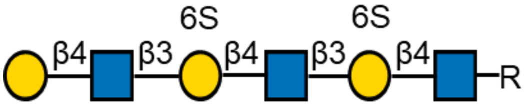

11 was prepared from **10** (0.8 mg, 0.7 μmol) using the general procedure of reduction reaction of Gal-6-aldehyde with sodium borohydride. After P2 purification, **8** was obtained as a white solid (0.8 mg, 98%). NMR data is confirmed with reference.

Compound 12

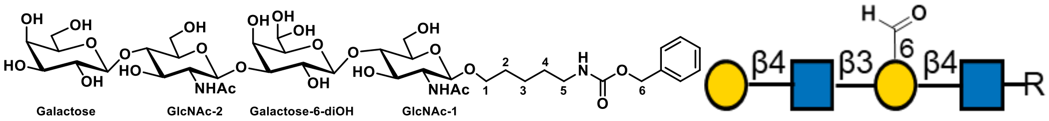

12 was prepared from **5** (10.0 mg, 12.2 μmol) using the general procedure for installation of β1,4 Gal using B4GalT1. After P2 purification, **6** was obtained as a white solid (11.5 mg, 96%).

^{1}H (600 MHz, D_2O): δ (ppm)

	H-1	H-2	H-3	H-4	H-5	H-6	NHAc
GlcNAc-1	4.51	3.71	3.68	3.67	3.59	3.98, 3.82	2.00 (s, 3H)
Galactose-6-diOH	4.47	3.61	3.73	4.32 (d, J = 3.2 Hz, 1H)	3.45 (d, J = 7.4 Hz, 1H)	5.14 (d, J = 7.3 Hz, 1H)	-
GlcNAc-2	4.72 (d, J = 8.4 Hz, 1H)	3.82	3.73	3.74	3.59	3.96, 3.85	2.04 (s, 3H)
Galactose	4.48	3.55	3.68	3.93 (d, J = 3.4 Hz, 1H)	3.73	3.76 (2H)	-

^{13}C (150 MHz, D_2O): δ (ppm)

	C-1	C-2	C-3	C-4	C-5	C-6	NHAc
GlcNAc-1	100.9	55.0	72.6	79.4	74.6	60.1	22.1
Galactose-6-diOH	103.2	69.8	82.2	67.8	76.7	88.1	-
GlcNAc-2	102.9	55.3	72.3	78.1	74.6	59.9	22.2
Galactose	103.0	71.1	72.6	68.7	75.4	61.0	-

Linker	1	2	3	4	5	6
H	3.87,3.57	1.59 – 1.52 (m, 2H)	1.35 – 1.28 (m, 2H)	1.49 (p, J = 7.3 Hz, 2H)	3.12 (t, J = 6.8 Hz, 2H)	5.11 (s, 2H)
C	70.4	28.2	22.4	28.5	40.3	66.7

HRMS (ESI-MS): m/z calculated for $C_{41}H_{65}N_3O_{24}$ Na $[M+Na]^+$: 1006.3851; found: 1006.3897.

Compound 13

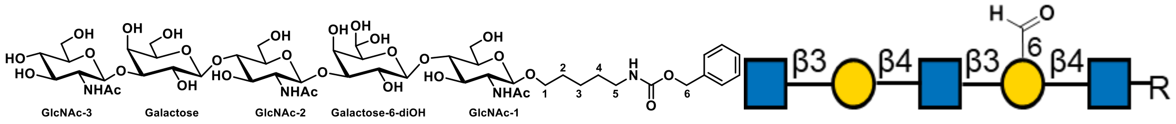

13 was prepared from **12** (9.6 mg, 9.8 μmol) using the general procedure for installation of β1,3 GlcNAc using B3GnT2. After P2 purification, **13** was obtained as a white solid (11.6 mg, quant).

^{1}H (600 MHz, D_2O): δ (ppm)

	H-1	H-2	H-3	H-4	H-5	H-6	NHAc
GlcNAc-1	4.51 (d, J = 6.9 Hz, 1H)	3.72	3.70	3.67	3.58	3.98, 3.83	2.00 (s, 3H)
Galactose-6-diOH	4.46	3.61	3.73	4.32 (d, J = 3.0 Hz, 1H)	3.45	5.13 (d, J = 7.3 Hz, 1H)	-
GlcNAc-2	4.72 (d, J = 8.4 Hz, 1H)	3.81	3.73	3.73	3.60	3.97, 3.82	2.06 – 2.02 (m, 6H)
Galactose	4.48	3.60	3.73	4.16 (d, J = 3.1 Hz, 1H)	3.72	3.76 (2H)	-
GlcNAc-3	4.69 (d, J = 8.4 Hz, 1H)	3.77	n/a	3.57	3.46	3.90, 3.76	2.06 – 2.02 (m, 6H)

^{13}C (150 MHz, D_2O): δ (ppm)

	C-1	C-2	C-3	C-4	C-5	C-6	NHAc
GlcNAc-1	101.0	55.0	72.3	79.5	74.5	60.1	22.1
Galactose-6-diOH	103.0	69.8	82.1	67.8	76.4	87.9	-
GlcNAc-2	102.7	55.2	72.2	78.1	74.8	60.1	22.1
Galactose	103.0	69.8	82.1	68.4	75.0	61.0	-
GlcNAc-3	103.0	55.5	n/a	n/a	n/a	60.5	22.1

Linker	1	2	3	4	5	6
H	3.87,3.57	1.59 – 1.52 (m, 2H)	1.35 – 1.28 (m, 2H)	1.49 (p, J = 7.3 Hz, 2H)	3.12 (t, J = 6.8 Hz, 2H)	5.11 (s, 2H)
C	70.5	28.3	22.3	28.6	40.5	66.7

HRMS (ESI-MS): m/z calculated for $C_{49}H_{78}N_4O_{29}$ Na $[M+Na]^+$: 1209.4644; found: 1209.4687.

Compound 14

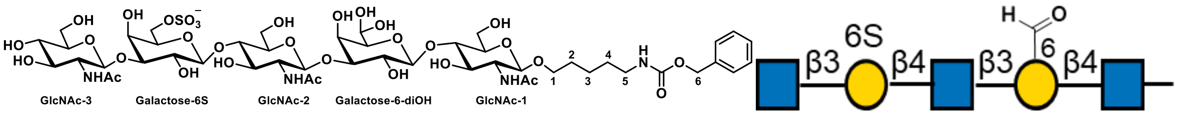

14 was prepared from **13** (2.5 mg, 2.1 μmol) using the general procedure for 6-O-sulfate installation of internal Galactose using CHST1. After P6 purification, **14** was obtained as a white solid (2.5 mg, 93%).

^{1}H (600 MHz, D_2O): δ (ppm)

	H-1	H-2	H-3	H-4	H-5	H-6	NHAc
GlcNAc-1	4.50	3.70	3.70	3.67	3.59	3.98, 3.83	2.01 (s, 3H)
Galactose-6-diOH	4.47 (d, J = 7.9 Hz, 1H)	3.62	3.74	4.32 (d, J = 3.2 Hz, 1H)	3.46	5.14 (d, J = 7.4 Hz, 1H)	-
GlcNAc-2	4.72 (d, J = 8.0 Hz, 1H)	3.83	3.76	3.72	3.60	3.98, 3.83	2.04 (s, 6H)
Galactose-6S	4.52 (d, J = 7.9 Hz, 1H)	3.59	3.75	4.23 (d, J = 3.3 Hz, 1H)	3.98	4.22 – 4.18 (m, 2H)	-
GlcNAc-3	4.69 (d, J = 8.4 Hz, 1H)	3.78	n/a	3.57	3.45	3.90, 3.77	2.04 (s, 6H)

^{13}C (150 MHz, D_2O): δ (ppm)

	C-1	C-2	C-3	C-4	C-5	C-6	NHAc
GlcNAc-1	100.1	55.1	72.6	79.6	74.7	60.1	22.3
Galactose-6-diOH	103.2	69.9	82.1	67.7	76.2	88.0	-
GlcNAc-2	102.8	55.3	72.1	79.1	74.8	60.1	22.3
Galactose-6S	102.9	69.6	82.3	68.0	72.8	67.4	-
GlcNAc-3	102.9	55.6	n/a	n/a	75.9	60.5	22.3

Linker	1	2	3	4	5	6
H	3.88,3.57	1.59 – 1.52 (m, 2H)	1.35 – 1.28 (m, 2H)	1.49 (p, J = 7.3 Hz, 2H)	3.12 (t, J = 6.8 Hz, 2H)	5.12 (s, 2H)
C	70.8	28.3	22.5	28.7	40.7	66.8

HRMS (ESI-MS): m/z calculated for $C_{49}H_{76}N_4O_{32}S$ [M-H]$^-$: 1265.4247; found: 1265.4148.

Compound 15

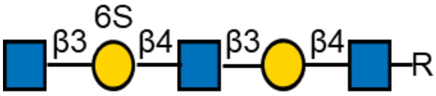

15 was prepared from **14** (1.0 mg, 0.8 μmol) using the general procedure of reduction reaction of Gal-6-aldehyde with sodium borohydride. After P6 purification, **15** was obtained as a white solid (0.9 mg, 87%).

^{1}H (600 MHz, D_2O): δ (ppm)

	H-1	H-2	H-3	H-4	H-5	H-6	NHAc
GlcNAc-1	4.50	3.70	3.69	3.70	3.60	3.98, 3.83	2.01 (s, 3H)
Galactose	4.46 (d, J = 8.1 Hz, 1H)	3.60	3.74	4.16	3.72	3.76	-
GlcNAc-2	4.72	3.81	3.77	3.72	3.60	3.98, 3.83	2.08 – 2.02 (m, 6H)
Galactose-6S	4.52	3.59	3.76	4.23	3.98	4.21 – 4.18 (m, 2H)	-
GlcNAc-3	4.69	3.78	n/a	3.57	3.46	3.90, 3.77	2.08 – 2.02 (m, 6H)

^{13}C (150 MHz, D_2O): δ (ppm)

	C-1	C-2	C-3	C-4	C-5	C-6	NHAc
GlcNAc-1	100.1	55.0	72.5	78.7	74.6	60.2	22.2
Galactose	103.0	69.9	82.3	68.4	75.0	60.7	-
GlcNAc-2	102.8	55.3	72.2	79.1	74.6	60.2	22.2
Galactose-6S	103.0	69.7	82.2	68.1	72.5	67.2	-
GlcNAc-3	103.2	55.7	n/a	n/a	75.7	60.5	22.2

Linker	1	2	3	4	5	6
H	3.88,3.57	1.59 – 1.52 (m, 2H)	1.35 – 1.28 (m, 2H)	1.49 (p, J = 7.3 Hz, 2H)	3.12 (t, J = 6.8 Hz, 2H)	5.12 (s, 2H)
C	70.5	28.3	22.4	28.6	40.5	66.8

HRMS (ESI-MS): m/z calculated for $C_{49}H_{76}N_4O_{31}S$ $[M-H]^-$: 1249.4297; found: 1249.4372.

Compound 16

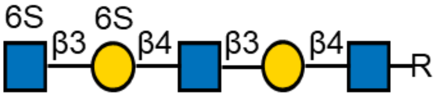

16 was prepared from **15** (0.9 mg, 0.7 μmol) using the general procedure for 6-O-sulfate installation of terminal GlcNAc using CHST2. After P6 purification, **16** was obtained as a white solid (0.8 mg, 85%).

^{1}H (600 MHz, D_2O): δ (ppm)

	H-1	H-2	H-3	H-4	H-5	H-6	NHAc
GlcNAc-1	4.51	3.71	3.69	3.70	3.59	3.98, 3.83	2.01 (s, 3H)
Galactose	4.46 (d, J = 7.9 Hz, 1H)	3.60	3.73	4.16 (d, J = 3.3 Hz, 1H)	3.72	3.76 (2H)	-
GlcNAc-2	4.71	3.81	3.77	3.72	3.59	3.98, 3.83	2.04 (s, 6H)
Galactose-6S	4.52	3.61	3.74	4.24	3.98	4.21 (2H)	-
GlcNAc-6S	4.72	3.79	3.59	3.54	3.67	4.34 (d, J = 10.7 Hz, 1H), 4.28 – 4.24 (m, 1H)	2.04 (s, 6H)

^{13}C (150 MHz, D_2O): δ (ppm)

	C-1	C-2	C-3	C-4	C-5	C-6	NHAc
GlcNAc-1	100.3	55.0	72.6	78.8	74.6	60.0	22.2
Galactose	102.8	69.8	82.2	68.3	75.0	61.0	-
GlcNAc-2	102.9	55.4	72.2	79.1	74.6	60.0	22.2
Galactose-6S	102.9	69.8	82.2	68.7	72.6	67.7	-
GlcNAc-6S	102.8	55.7	73.9	69.3	73.9	66.9	22.2

Linker	1	2	3	4	5	6
H	3.88,3.57	1.59 – 1.52 (m, 2H)	1.35 – 1.28 (m, 2H)	1.49 (p, J = 7.3 Hz, 2H)	3.12 (t, J = 6.8 Hz, 2H)	5.12 (s, 2H)
C	70.5	28.3	22.4	28.6	40.5	66.8

HRMS (ESI-MS): m/z calculated for $C_{49}H_{76}N_4O_{34}S_2$ $[M-2H]^{2-}$: 664.1896; found: 664.1841.

Compound 17

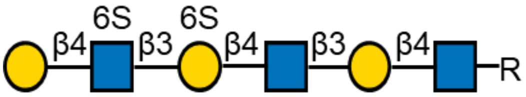

17 was prepared from **16** (0.8 mg, 0.6 μmol) using the general procedure for installation of β1,4 Gal using B4GalT4. After P6 purification, **17** was obtained as a white solid (0.9 mg, 99%). NMR data is confirmed with reference.

Compound 18

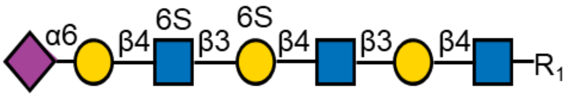

R_1= $O(CH_2)_5NHCbz$

18 was prepared from **17** (0.9 mg, 0.6 μmol) using the general procedure for installation of terminal α2,6 Neu5Ac using ST6Gal1. After P6 purification, **18** was obtained as a white solid (0.8 mg, 74%).

^{1}H (600 MHz, D_2O): δ (ppm)

	H-1	H-2	H-3	H-4	H-5	H-6	H-7	H-8	H-9	NHAc
GlcNAc-1	4.51	3.72	3.69	3.69	3.58	3.98, 3.84	-	-	-	2.09 – 1.98 (m, 12H)
Galactose-1	4.47 (d, J = 7.9 Hz, 1H)	3.59	3.72	4.17	3.73	3.76 (2H)	-	-	-	-
GlcNAc-2	4.71 (d, J = 7.2 Hz, 1H)	3.81	3.73	3.71	3.63	3.98, 3.84	-	-	-	2.09 – 1.98 (m, 12H)
Galactose-6S	4.52	3.61	3.76	4.24	3.99	4.22 (2H)	-	-	-	-
GlcNAc-6S	4.77	3.84	n/a	3.82	3.85	4.42 (d, J = 10.8 Hz, 1H), 4.35 – 4.29 (m, 1H)	-	-	-	2.09 – 1.98 (m, 12H)
Galactose-2	4.49	3.54	3.69	3.94 (d, J = 3.0 Hz, 1H)	n/a	4.01, 3.55	-	-	-	-
Sialic acid	-	-	2.68 (dd, J = 12.5, 4.6 Hz, 1H), 1.74 (t, J = 12.2 Hz, 1H)	3.68	3.82	n/a	3.57	3.90	3.89, 3.65	2.09 – 1.98 (m, 12H)

^{13}C (150 MHz, D_2O): δ (ppm)

	C-1	C-2	C-3	C-4	C-5	C-6	C-7	C-8	C-9	NHAc
GlcNAc-1	100.6	54.9	72.5	78.8	74.9	60.1	-	-	-	22.2
Galactose-1	102.9	70.0	82.3	68.1	75.1	61.1	-	-	-	-
GlcNAc-2	102.7	55.2	72.7	79.1	74.6	60.1	-	-	-	22.2
Galactose-6S	102.5	70.1	82.3	68.2	72.8	67.9	-	-	-	-
GlcNAc-6S	102.5	55.3	n/a	77.3	72.8	66.8	-	-	-	22.2
Galactose-2	103.5	70.9	72.7	68.2	n/a	63.6	-	-	-	-
Sialic acid	n/a	n/a	40.1	n/a	51.5	n/a	68.6	72.1	62.7	22.2

Linker	1	2	3	4	5	6
H	3.87,3.57	1.59 – 1.52 (m, 2H)	1.35 – 1.28 (m, 2H)	1.53 – 1.45 (m, 2H)	3.12 (t, J = 6.5 Hz, 2H)	5.12 (s, 2H)
C	69.9	28.3	22.5	28.5	40.4	66.8

HRMS (ESI-MS): m/z calculated for $C_{66}H_{103}N_5O_{47}S_2$ $[M-2H]^{2-}$: 890.7638; found: 890.7596.

Compound 19

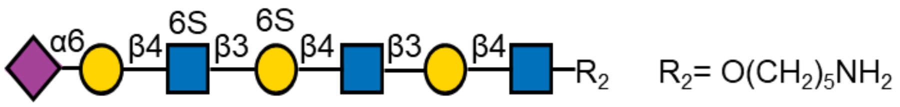

19 was prepared from **18** (0.4 mg, 0.25 μmol) using the general procedure for Cbz deprotection using $Pd(OH)_2$ reduction. After purification, **19** was obtained as a white solid (0.3 mg, 62%).

^{1}H (600 MHz, D_2O): δ (ppm)

	H-1	H-2	H-3	H-4	H-5	H-6	H-7	H-8	H-9	NHAc
GlcNAc-1	4.53	3.72	3.69	3.69	3.58	3.98, 3.84	-	-	-	2.08 – 2.02 (m, 12H)
Galactose-1	4.47 (d, J = 7.9 Hz, 1H)	3.60	3.74	4.17 (d, J = 3.2 Hz, 1H)	3.73	3.76 (2H)	-	-	-	-
GlcNAc-2	4.71 (d, J = 7.7 Hz, 1H)	3.81	3.73	3.71	3.63	3.98, 3.84	-	-	-	2.08 – 2.02 (m, 12H)
Galactose-6S	4.53	3.62	3.76	4.25	3.99	4.21 (2H)	-	-	-	-
GlcNAc-6S	4.77	3.84	n/a	n/a	3.84	4.42 (d, J = 11.0 Hz, 1H), 4.32 (dd, J = 11.1, 5.0 Hz, 1H)	-	-	-	2.08 – 2.02 (m, 12H)
Galactose-2	4.50 (d, J = 7.8 Hz, 1H)	3.55	3.69	3.94 (d, J = 3.1 Hz, 1H)	n/a	4.01, 3.55	-	-	-	-
Sialic acid	-	-	2.68 (dd, J = 12.4, 4.6 Hz, 1H), 1.73 (t, J = 12.1 Hz, 1H)	3.67	3.82	n/a	3.57	3.91	3.89, 3.65	2.08 – 2.02 (m, 12H)

^{13}C (150 MHz, D_2O): δ (ppm)

	C-1	C-2	C-3	C-4	C-5	C-6	C-7	C-8	C-9	NHAc
GlcNAc-1	100.9	54.9	72.4	78.7	74.9	60.0	-	-	-	22.2
Galactose-1	102.9	70.0	82.1	68.3	75.1	61.0	-	-	-	-

GlcNAc-2	102.7	55.1	72.7	79.1	74.7	60.0	-	-	-	22.2
Galactose-6S	102.6	69.7	82.1	68.1	72.9	67.9	-	-	-	-
GlcNAc-6S	102.6	55.2	n/a	n/a	73.3	66.8	-	-	-	22.2
Galactose-2	103.5	70.9	72.7	68.1	n/a	63.3	-	-	-	-
Sialic acid	n/a	n/a	40.1	n/a	52.0	n/a	68.5	72.5	62.7	22.2

Linker	1	2	3	4	5
H	3.90,3.71	1.61 (p, J = 6.9 Hz, 2H)	1.46 – 1.36 (m, 2H)	1.68 (p, J = 7.8 Hz, 2H)	3.02 – 2.96 (m, 2H)
C	69.8	28.1	22.1	26.7	39.4

HRMS (ESI-MS): m/z calculated for $C_{58}H_{97}N_5O_{45}S_2$ $[M-2H]^{2-}$: 823.7454; found: 823.7386.

4) References

1. Peng, W. J.; Pranskevich, J.; Nycholat, C.; Gilbert, M.; Wakarchuk, W.; Paulson, J. C.; Razi, N., *Helicobacter pylori* beta 1,3-*N*-acetylglucosaminyltransferase for versatile synthesis of type 1 and type 2 poly-LacNAcs on *N*-linked, *O*-linked and I-antigen glycans. *Glycobiology* **2012,** *22* (11), 1453-1464.
2. Sugiarto, G.; Lau, K.; Qu, J.; Li, Y.; Lim, S.; Mu, S.; Ames, J. B.; Fisher, A. J.; Chen, X., A sialyltransferase mutant with decreased donor hydrolysis and reduced sialidase activities for directly sialylating lewisx. *ACS Chem. Biol.* **2012,** *7* (7), 1232-1240.
3. Li, Y.; Xue, M.; Sheng, X.; Yu, H.; Zeng, J.; Thon, V.; Chen, Y.; Muthana, M. M.; Wang, P. G.; Chen, X., Donor substrate promiscuity of bacterial β1–3-N-acetylglucosaminyltransferases and acceptor substrate flexibility of β1–4-galactosyltransferases. *Biorg. Med. Chem.* **2016,** *24* (8), 1696-1705.
4. Prudden, A. R.; Liu, L.; Capicciotti, C. J.; Wolfert, M. A.; Wang, S.; Gao, Z.; Meng, L.; Moremen, K. W.; Boons, G. J., Synthesis of asymmetrical multiantennary human milk oligosaccharides. *Proc. Natl. Acad. Sci. U. S. A.* **2017,** *114* (27), 6954-6959.
5. Meng, L.; Forouhar, F.; Thieker, D.; Gao, Z.; Ramiah, A.; Moniz, H.; Xiang, Y.; Seetharaman, J.; Milaninia, S.; Su, M., Enzymatic basis for N-glycan sialylation: structure of rat α2, 6-sialyltransferase (ST6GAL1) reveals conserved and unique features for glycan sialylation. *J. Biol. Chem.* **2013,** *288* (48), 34680-34698.
6. Moremen, K. W.; Ramiah, A.; Stuart, M.; Steel, J.; Meng, L.; Forouhar, F.; Moniz, H. A.; Gahlay, G.; Gao, Z.; Chapla, D.; Wang, S.; Yang, J.-Y.; Prabhakar, P. K.; Johnson, R.; Rosa, M. d.; Geisler, C.; Nairn, A. V.; Seetharaman, J.; Wu, S.-C.; Tong, L.; Gilbert, H. J.; LaBaer, J.; Jarvis, D. L., Expression system for structural and functional studies of human glycosylation enzymes. *Nat. Chem. Biol.* **2018,** *14* (2), 156-162.
7. Wu, Y.; Vos, G. M.; Huang, C.; Chapla, D.; Kimpel, A. L. M.; Moremen, K. W.; de Vries, R. P.; Boons, G. J., Exploiting substrate specificities of 6-O-sulfotransferases to enzymatically synthesize keratan sulfate oligosaccharides. *JACS Au* **2023,** *3* (11), 3155-3164.
8. Wu, Y.; Bosman, G. P.; Chapla, D.; Huang, C.; Moremen, K. W.; de Vries, R. P.; Boons, G. J., A biomimetic synthetic strategy can provide keratan sulfate I and II oligosaccharides with diverse fucosylation and sulfation patterns. *J. Am. Chem. Soc.* **2024,** *146* (13), 9230-9240.

5) NMR Spectra

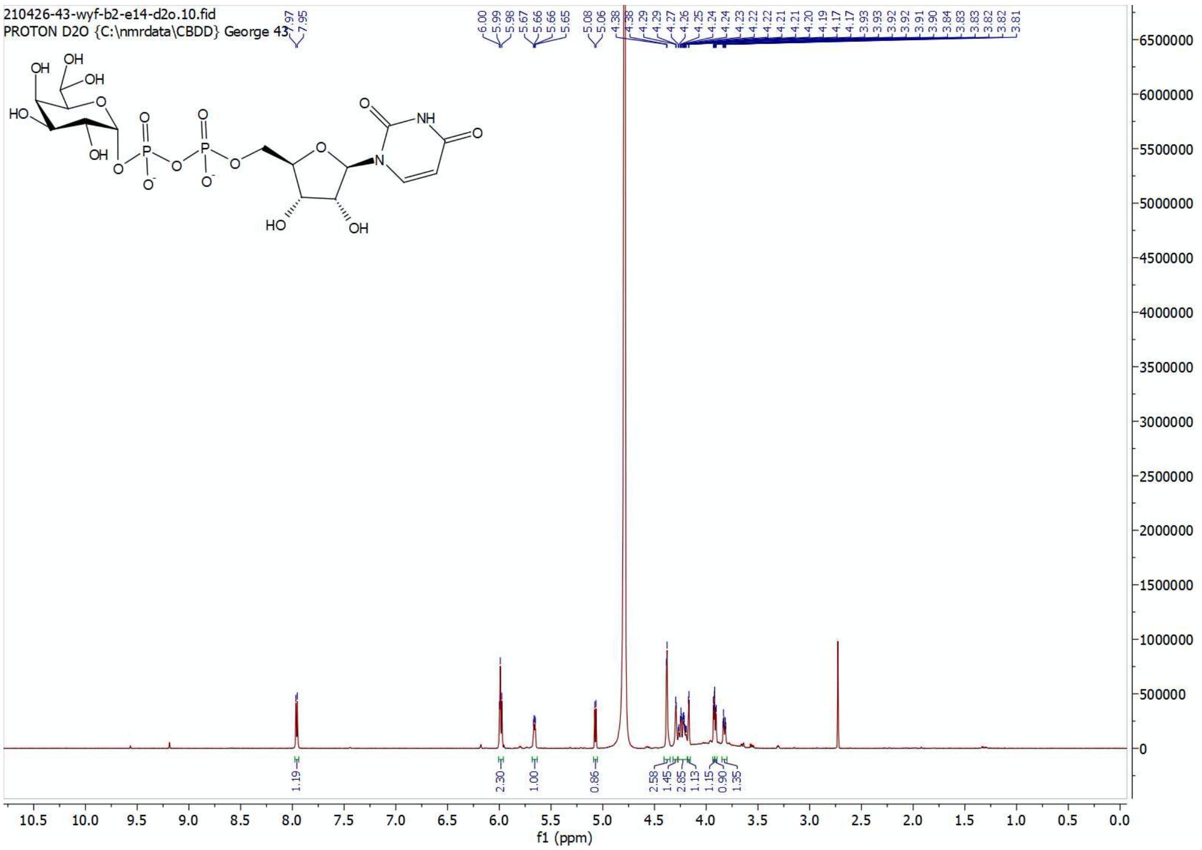

^{1}H NMR of 2; 600MHz; D_2O

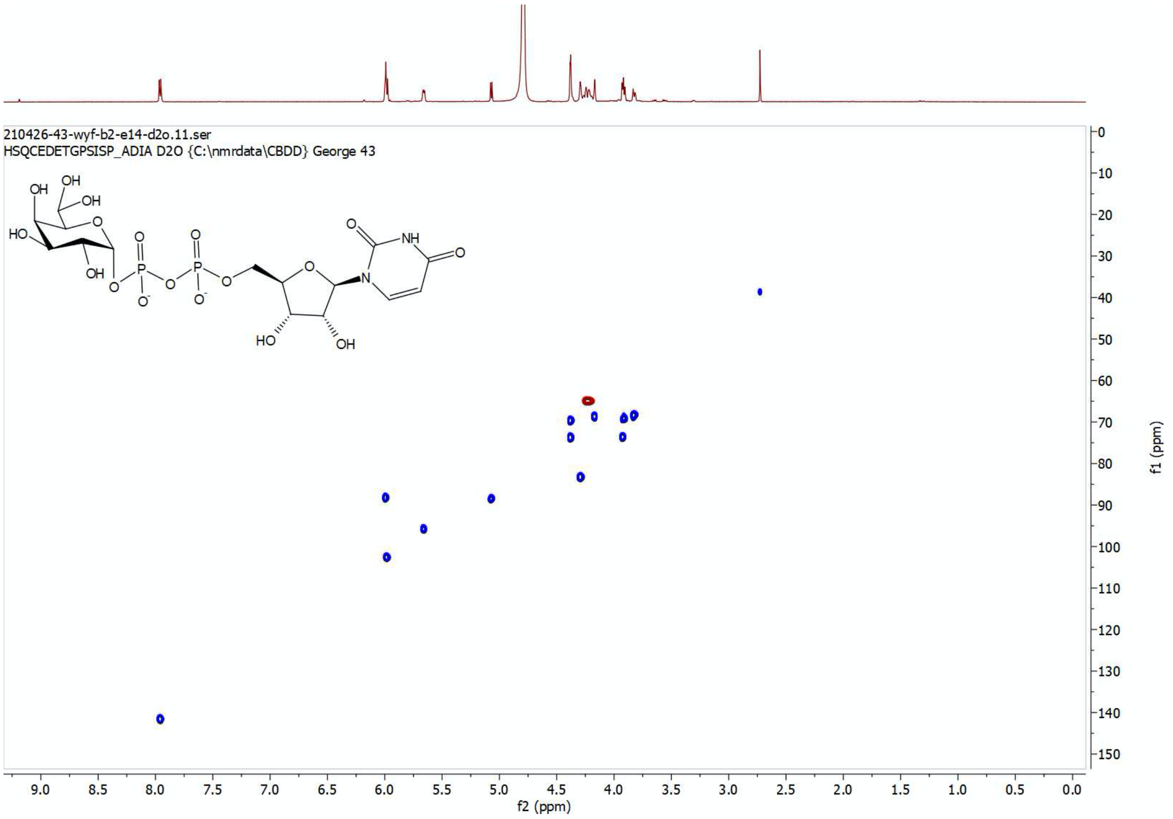

HSQC of 2; 600 MHz/150 MHz, D_2O

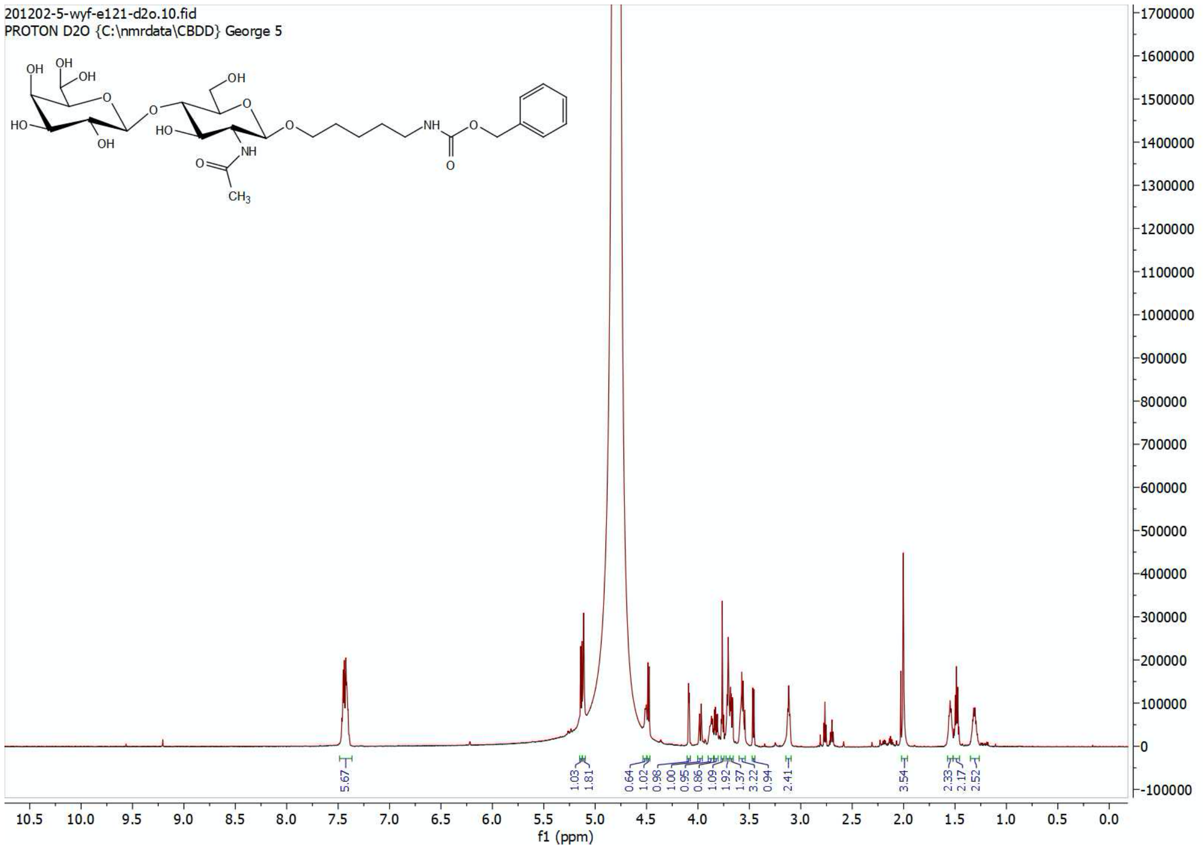

^{1}H NMR of 4; 600MHz; D_2O

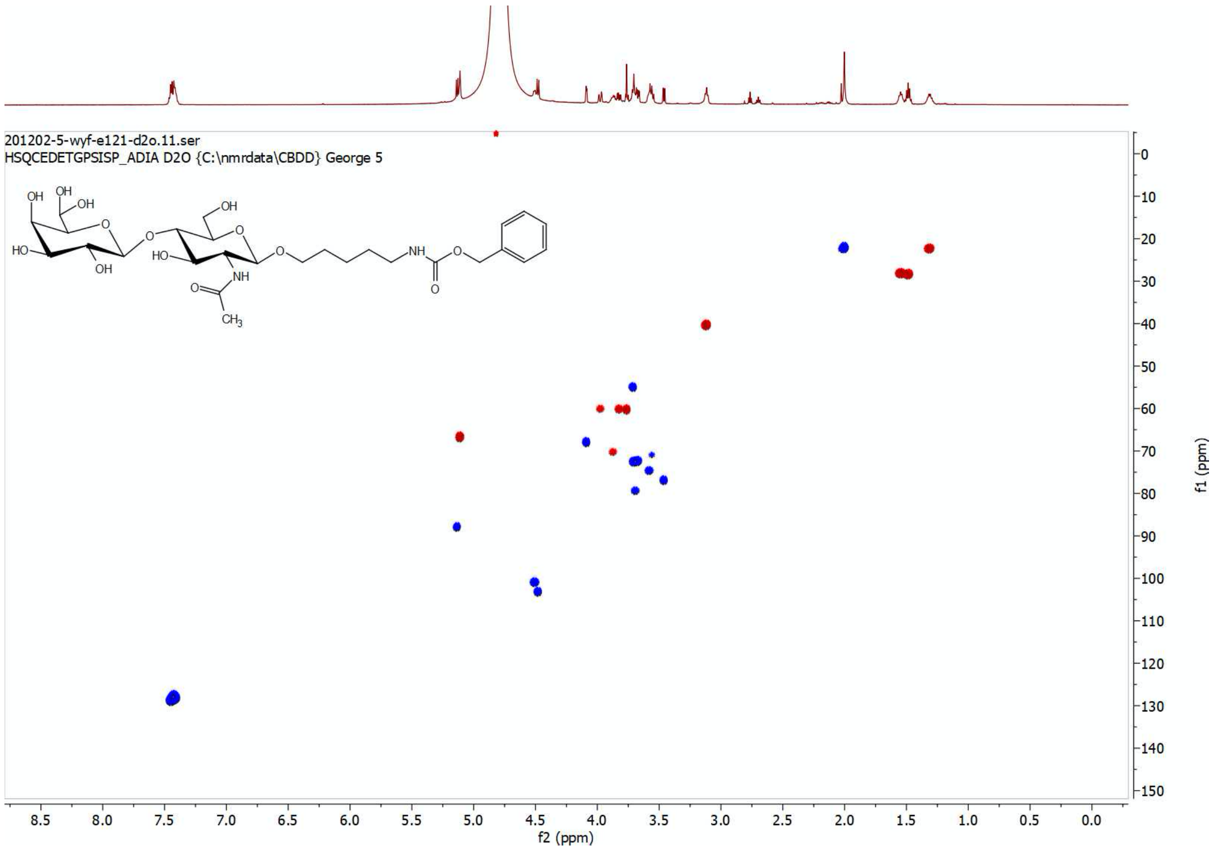

HSQC of 4; 600 MHz/150 MHz, D_2O

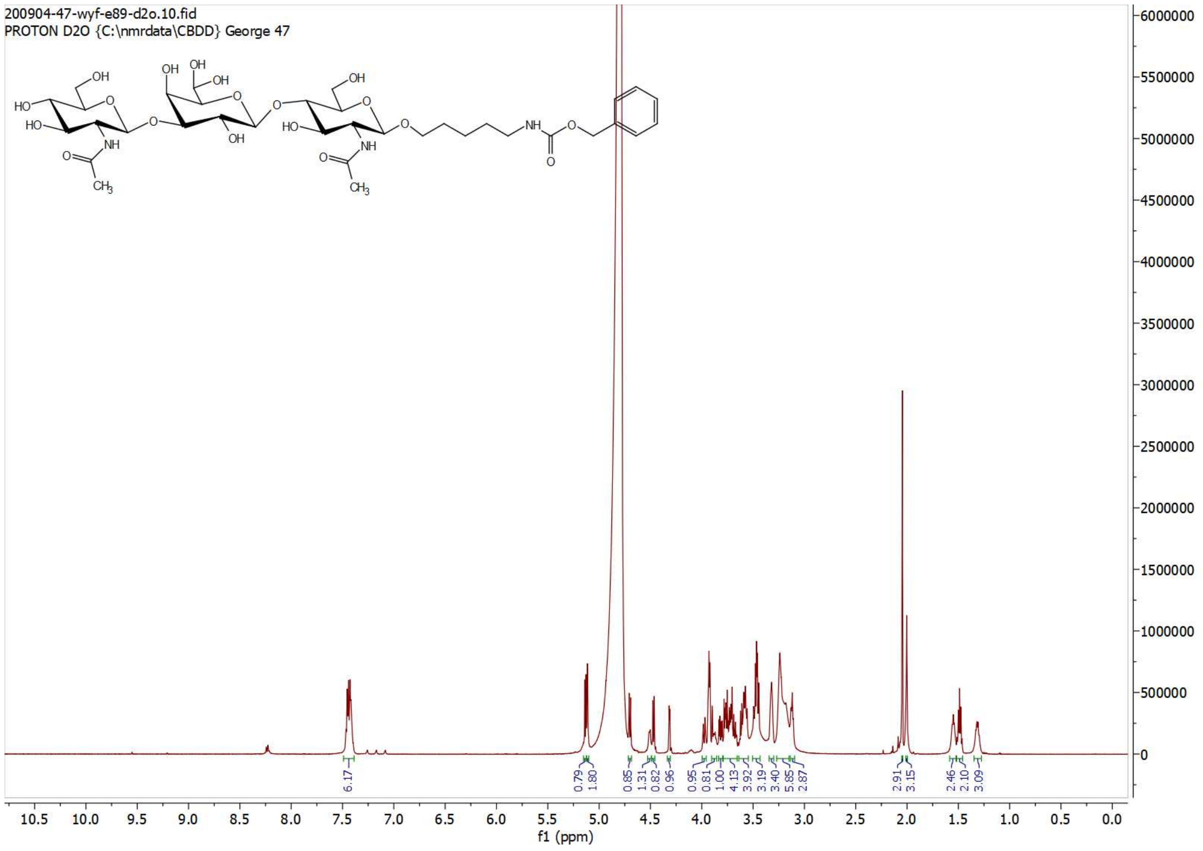

^{1}H NMR of 5; 600MHz; D_2O

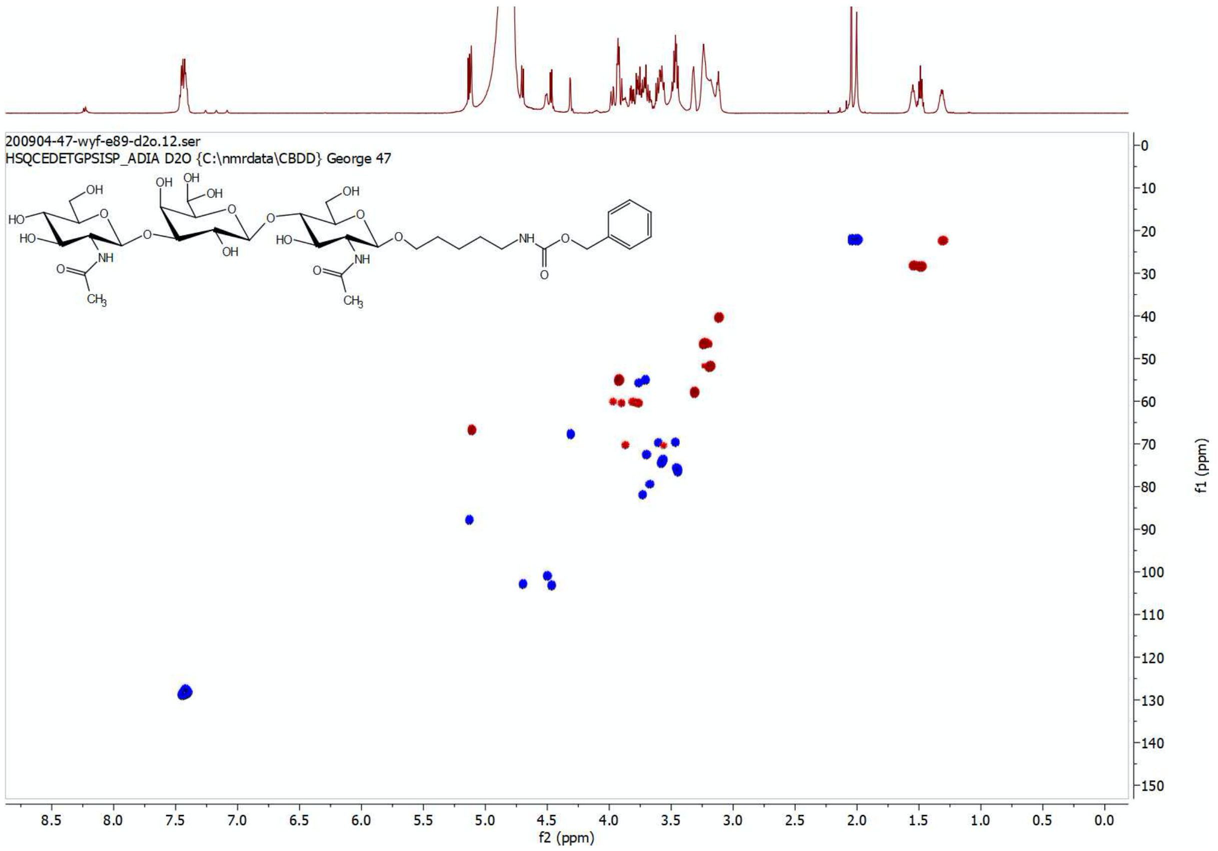

HSQC of 5; 600 MHz/150 MHz, D_2O

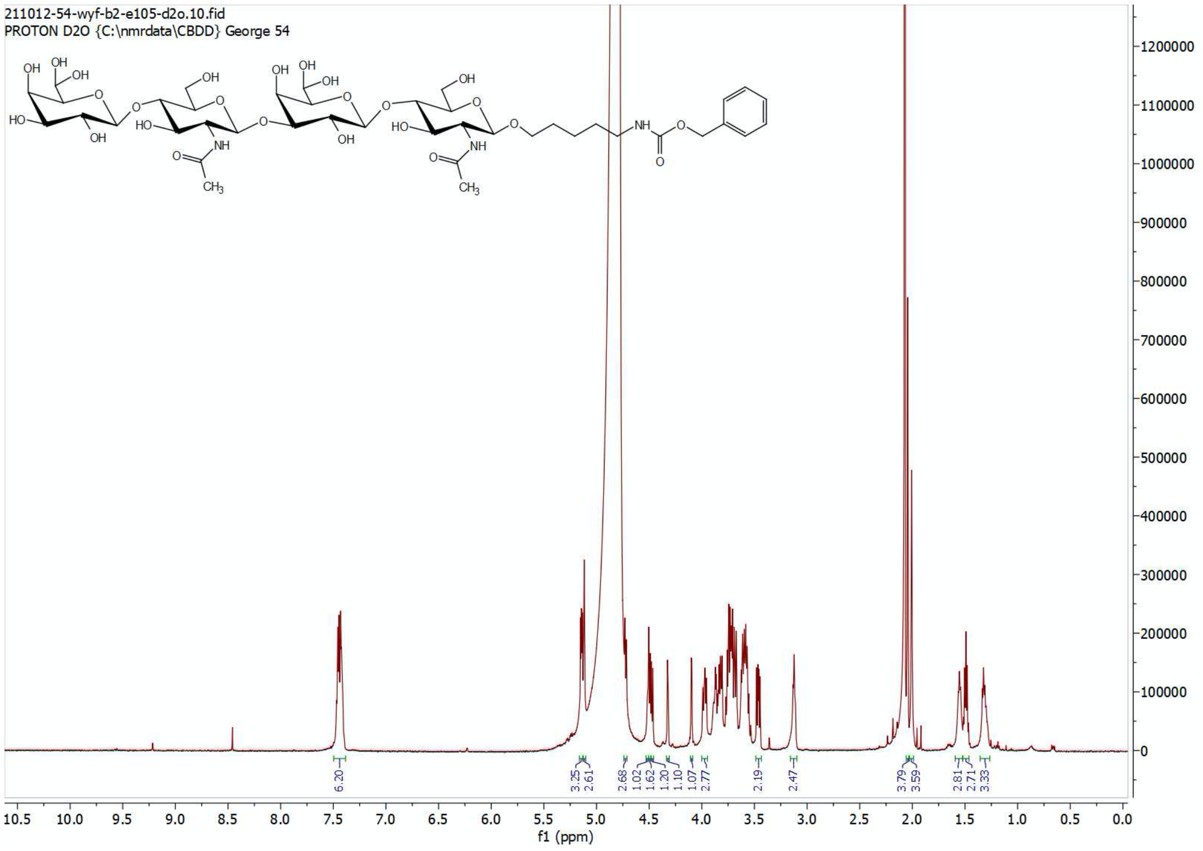

^{1}H NMR of 6; 600MHz; D_2O

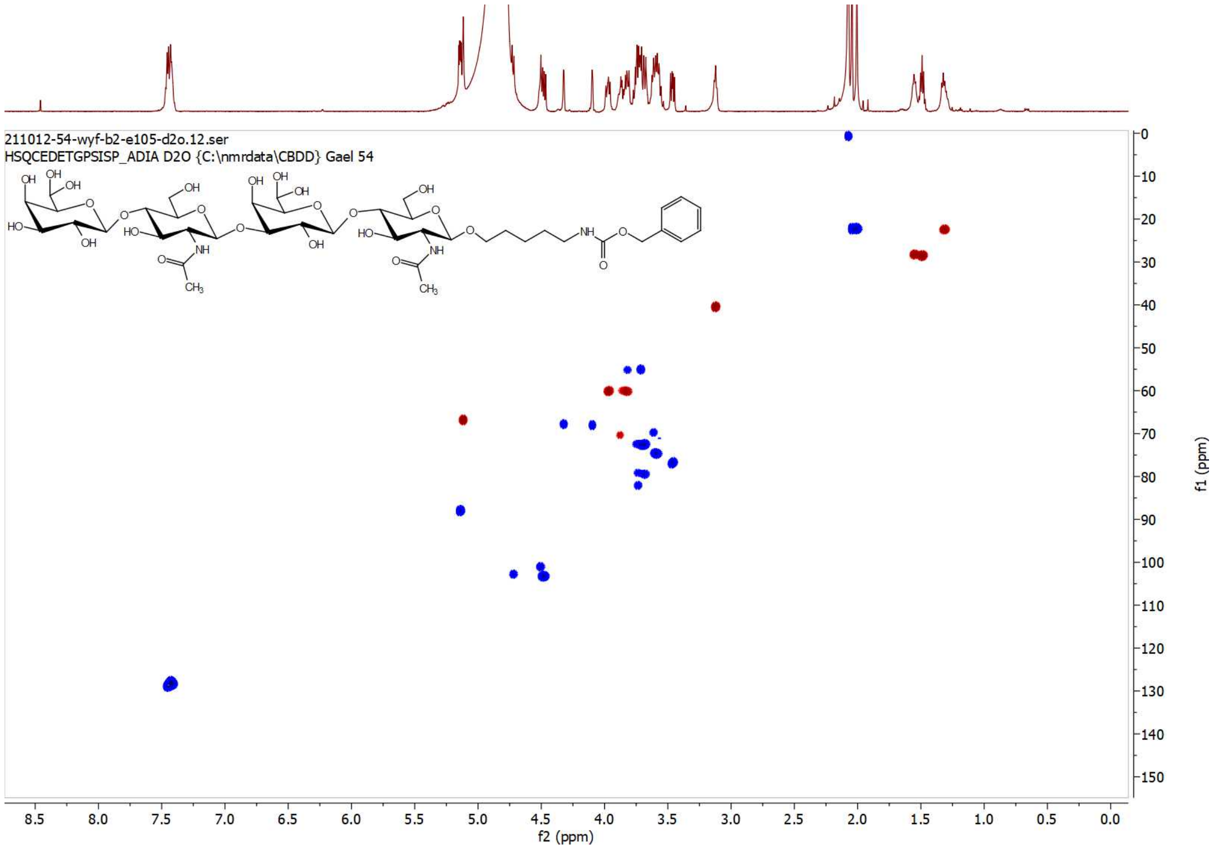

HSQC of 6; 600 MHz/150 MHz, D_2O

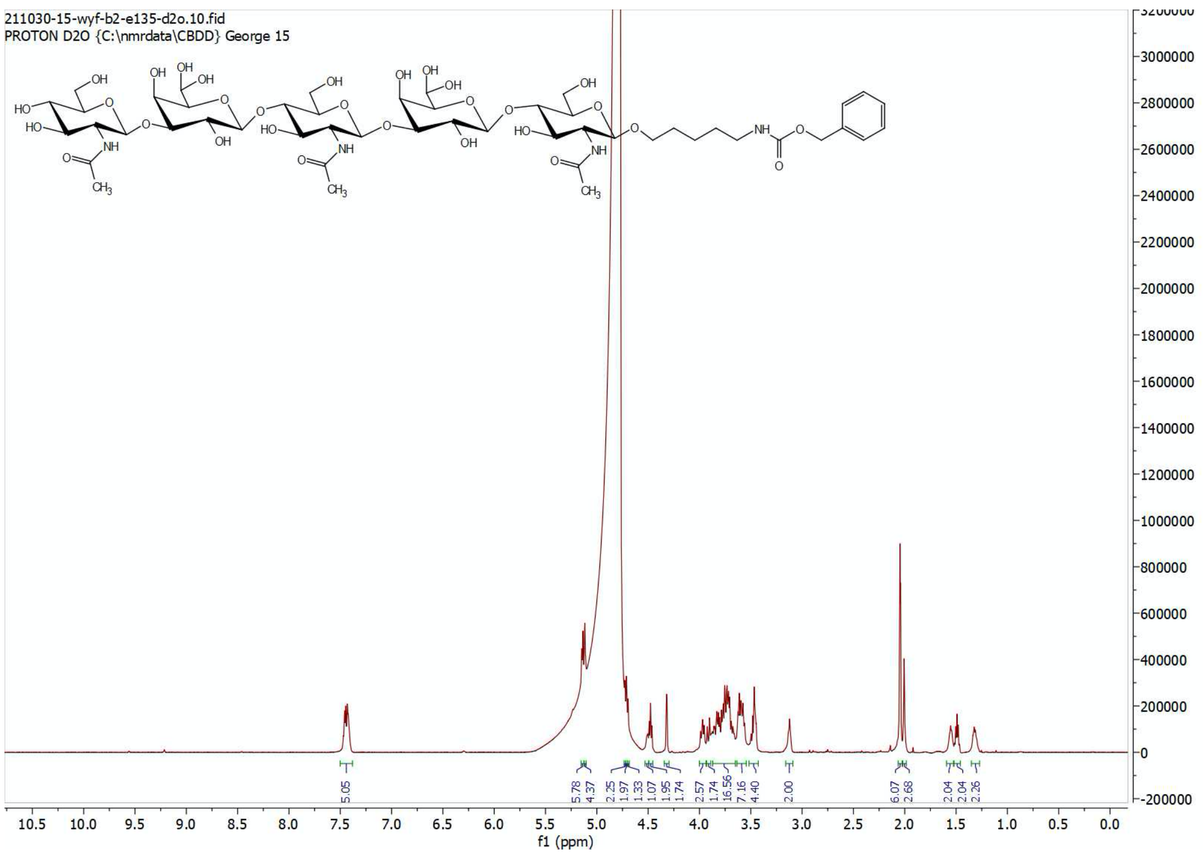

^{1}**H NMR of 7; 600MHz; D$_2$O**

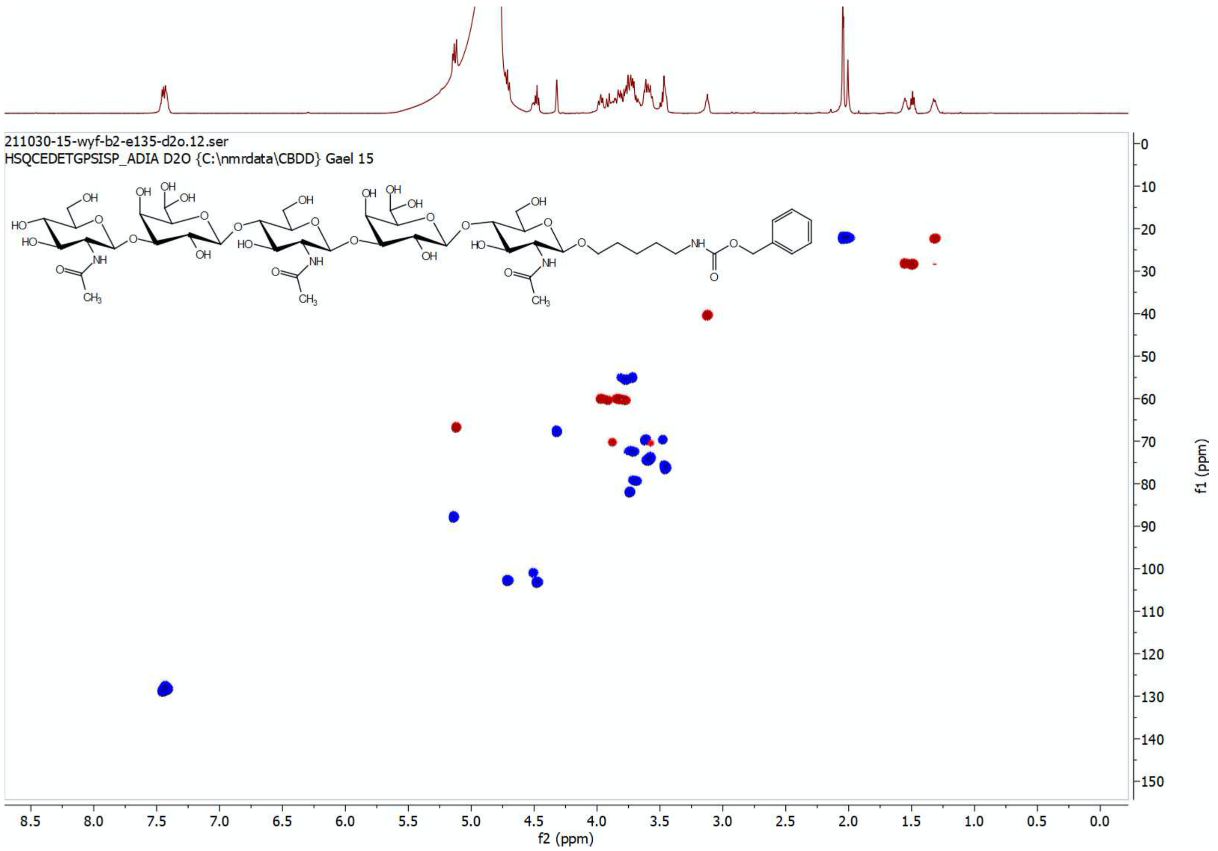

HSQC of 7; 600 MHz/150 MHz, D$_2$O

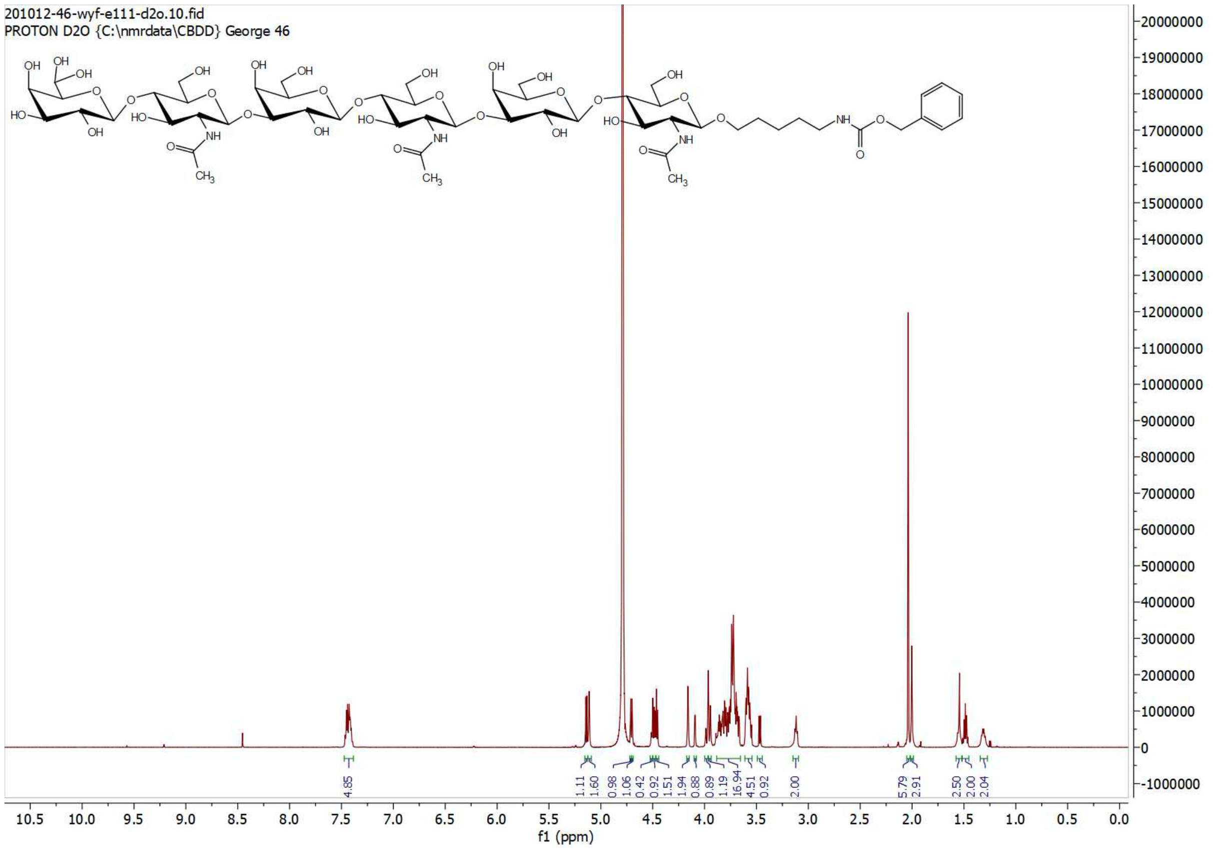

^{1}H NMR of 9; 600MHz; D_2O

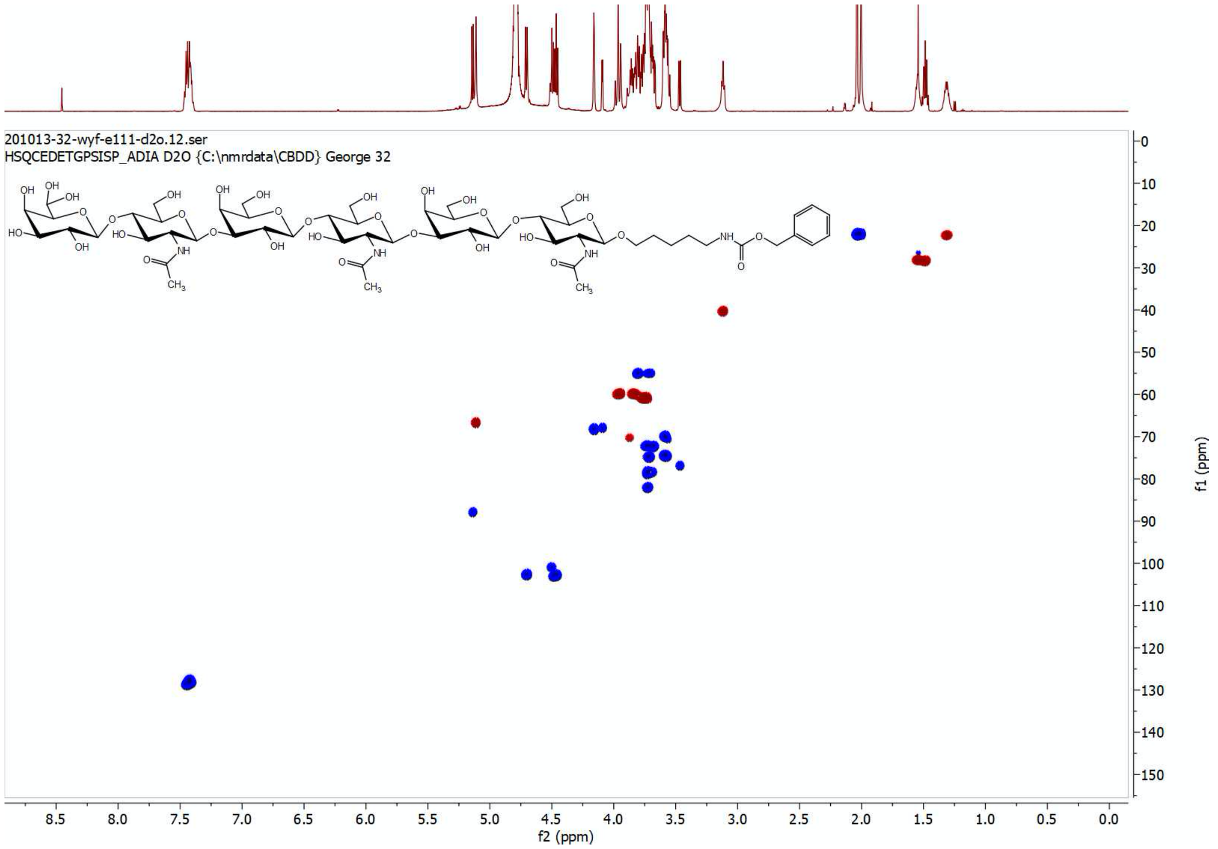

HSQC of 9; 600 MHz/150 MHz, D_2O

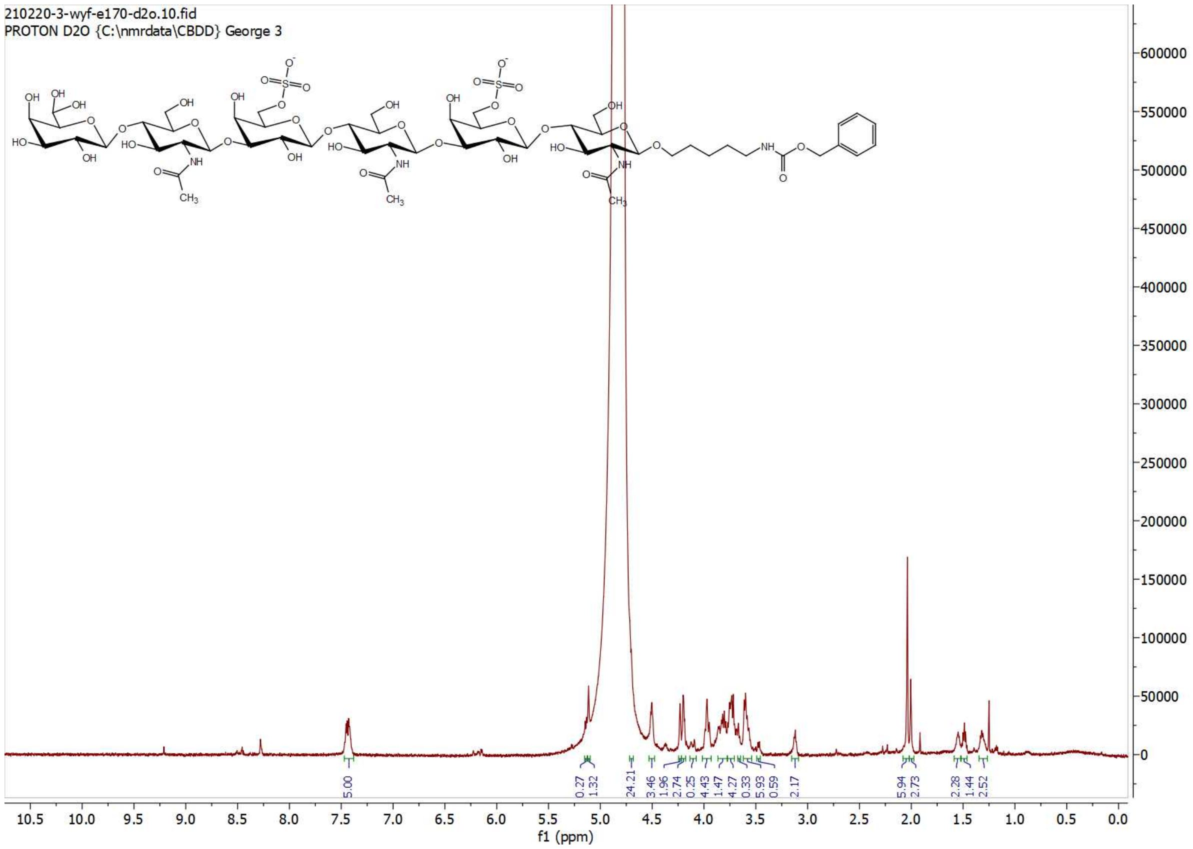

^{1}H NMR of 10; 600MHz; D_2O

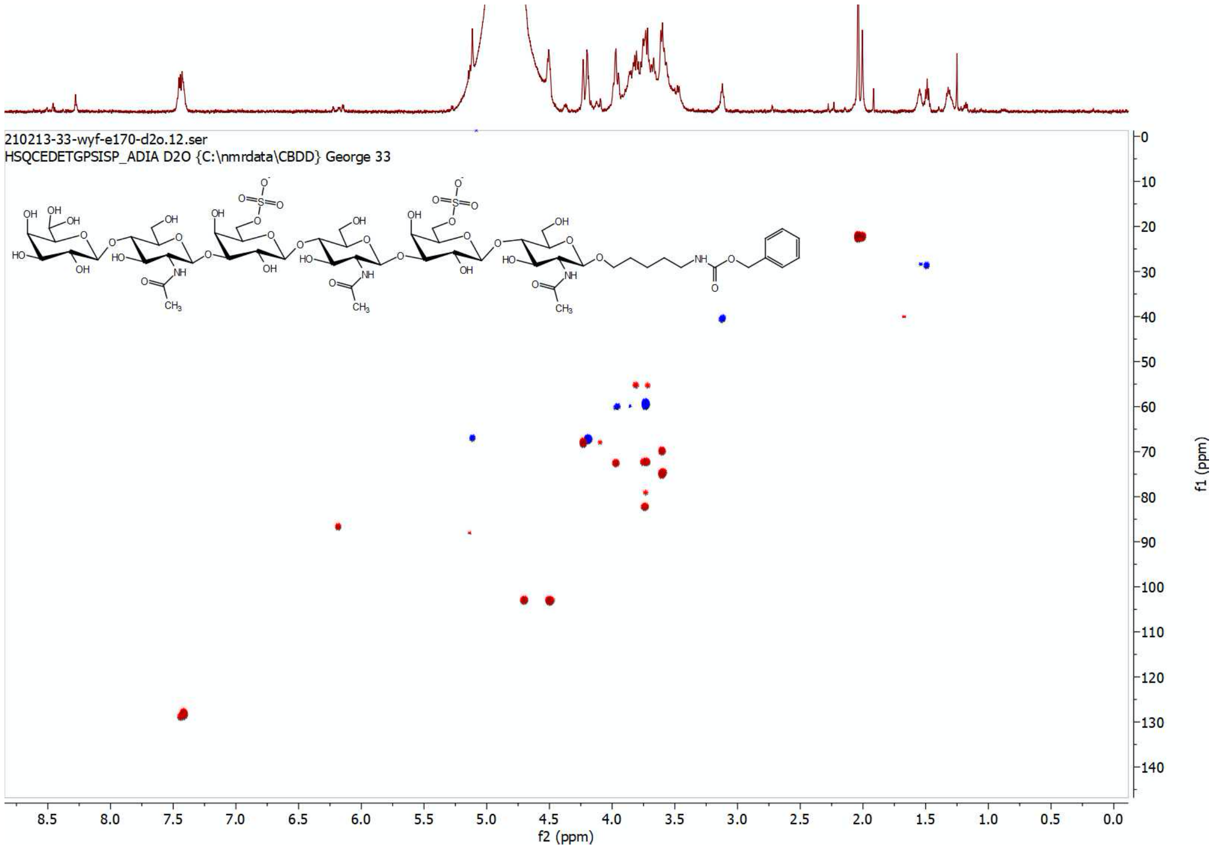

HSQC of 10; 600 MHz/150 MHz, D_2O

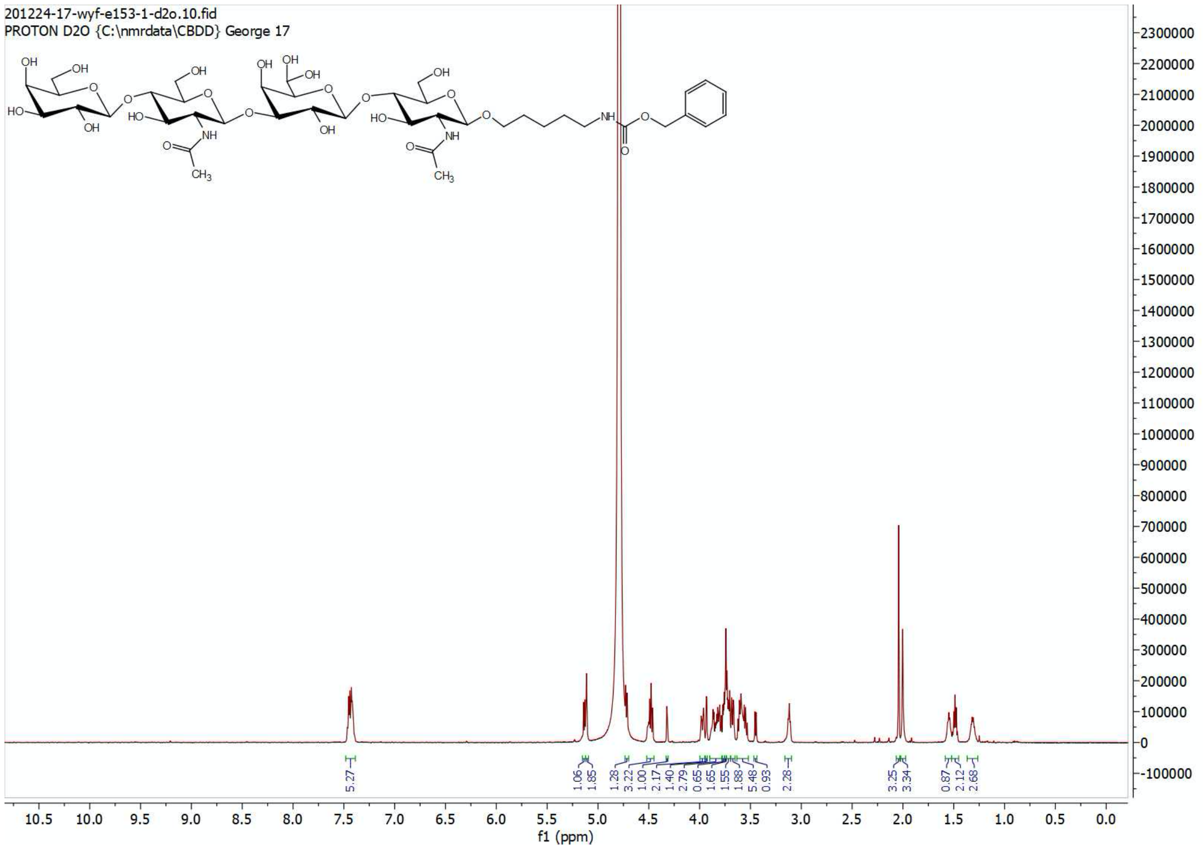

^{1}H NMR of 12; 600MHz; D_2O

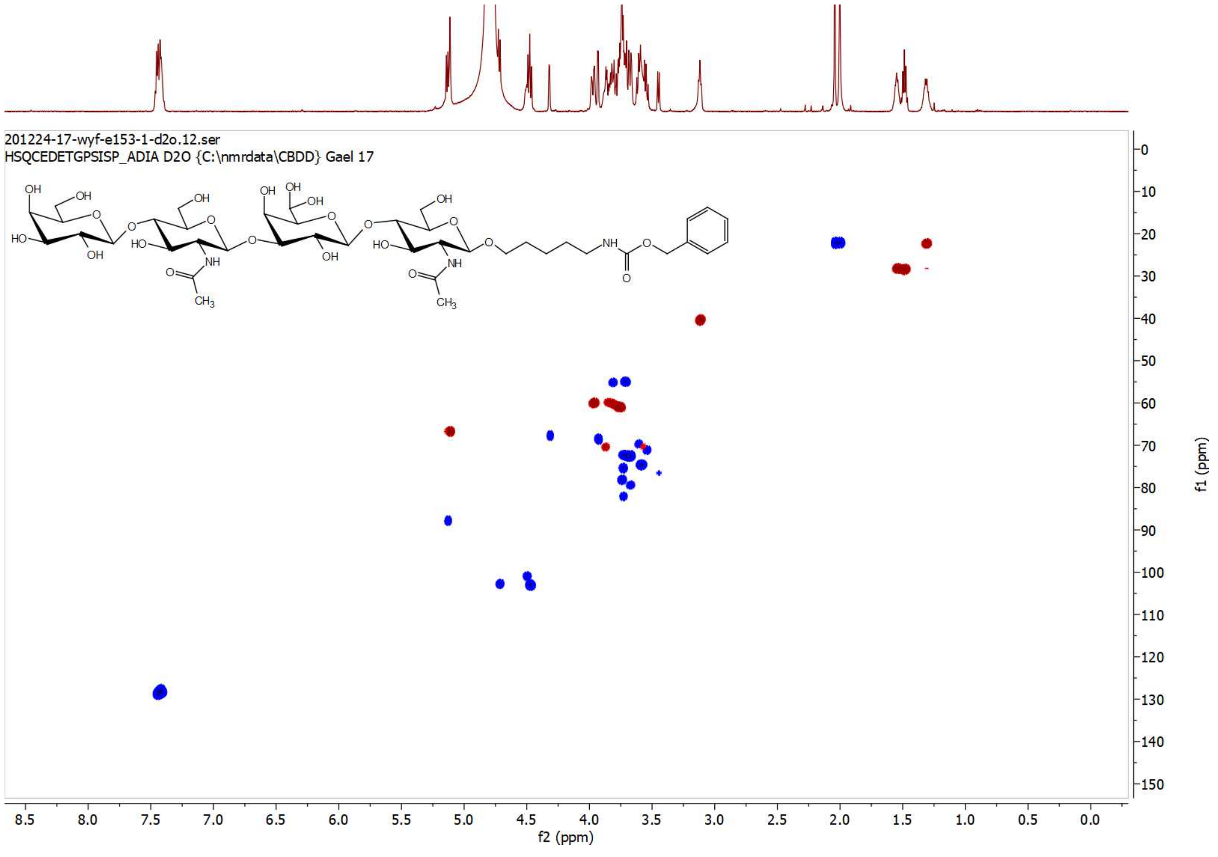

HSQC of 12; 600 MHz/150 MHz, D_2O

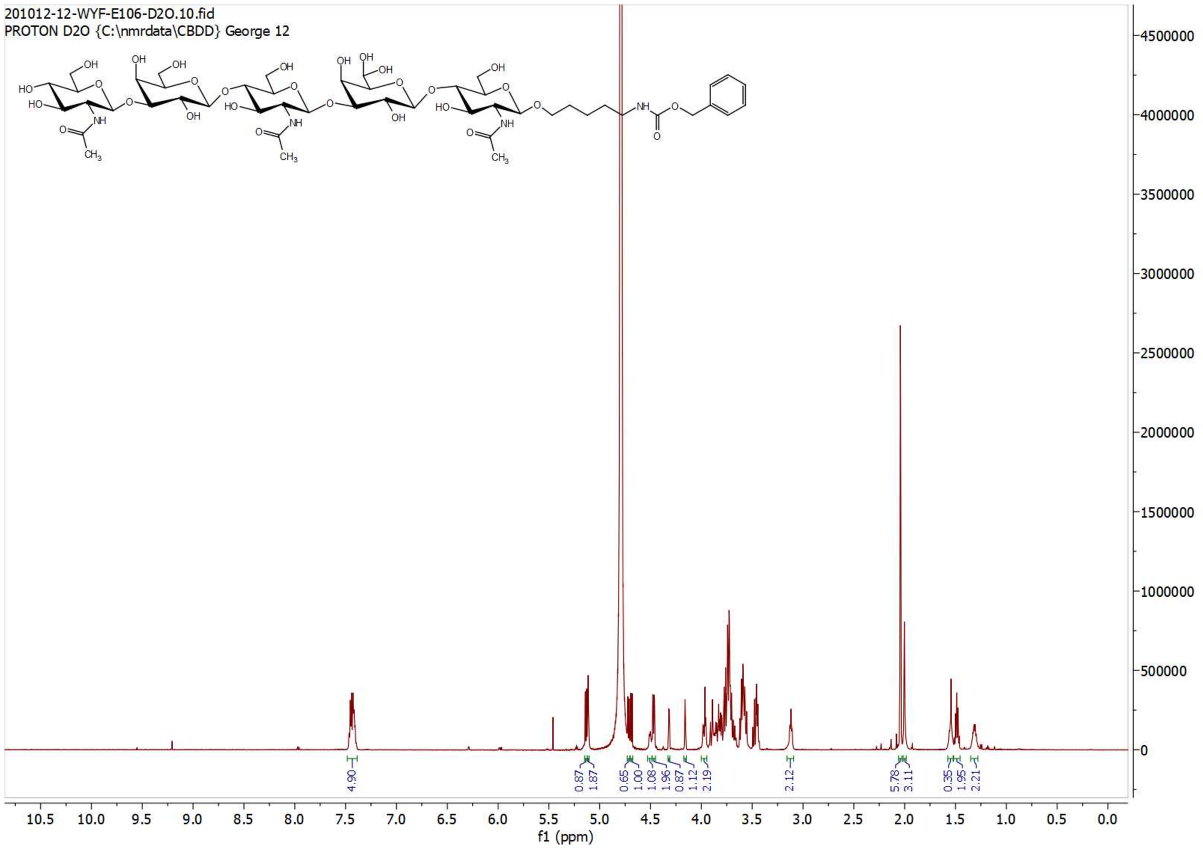

^{1}H NMR of 13; 600MHz; D_2O

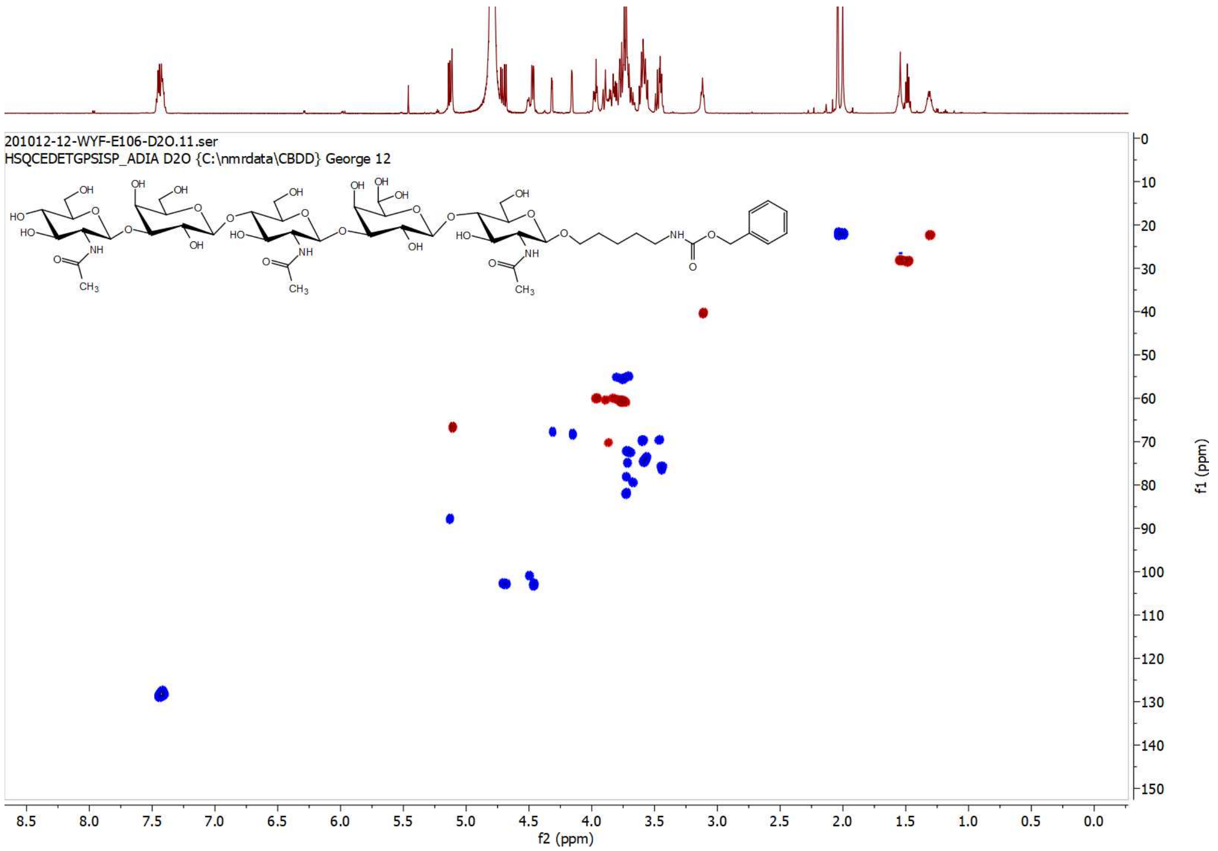

HSQC of 13; 600 MHz/150 MHz, D_2O

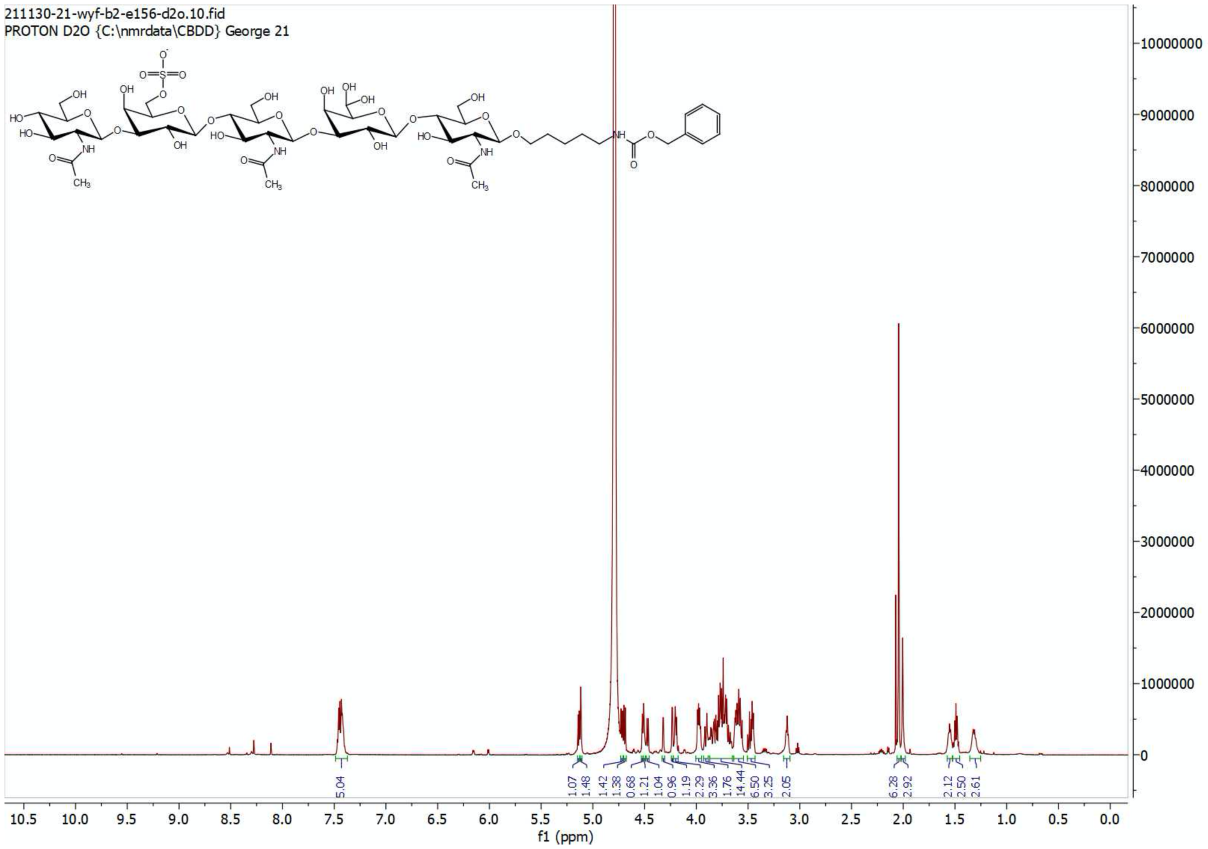

^{1}H NMR of 14; 600MHz; D_2O

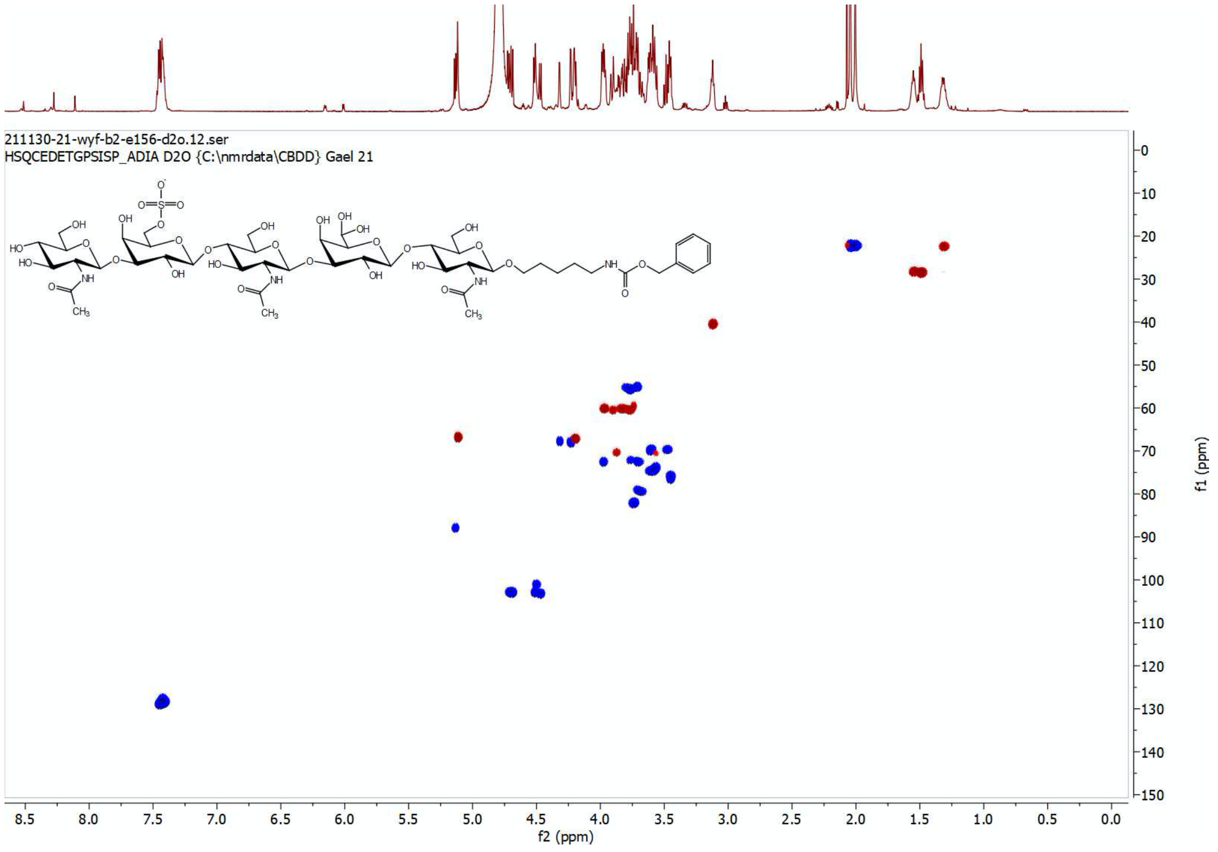

HSQC of 14; 600 MHz/150 MHz, D_2O

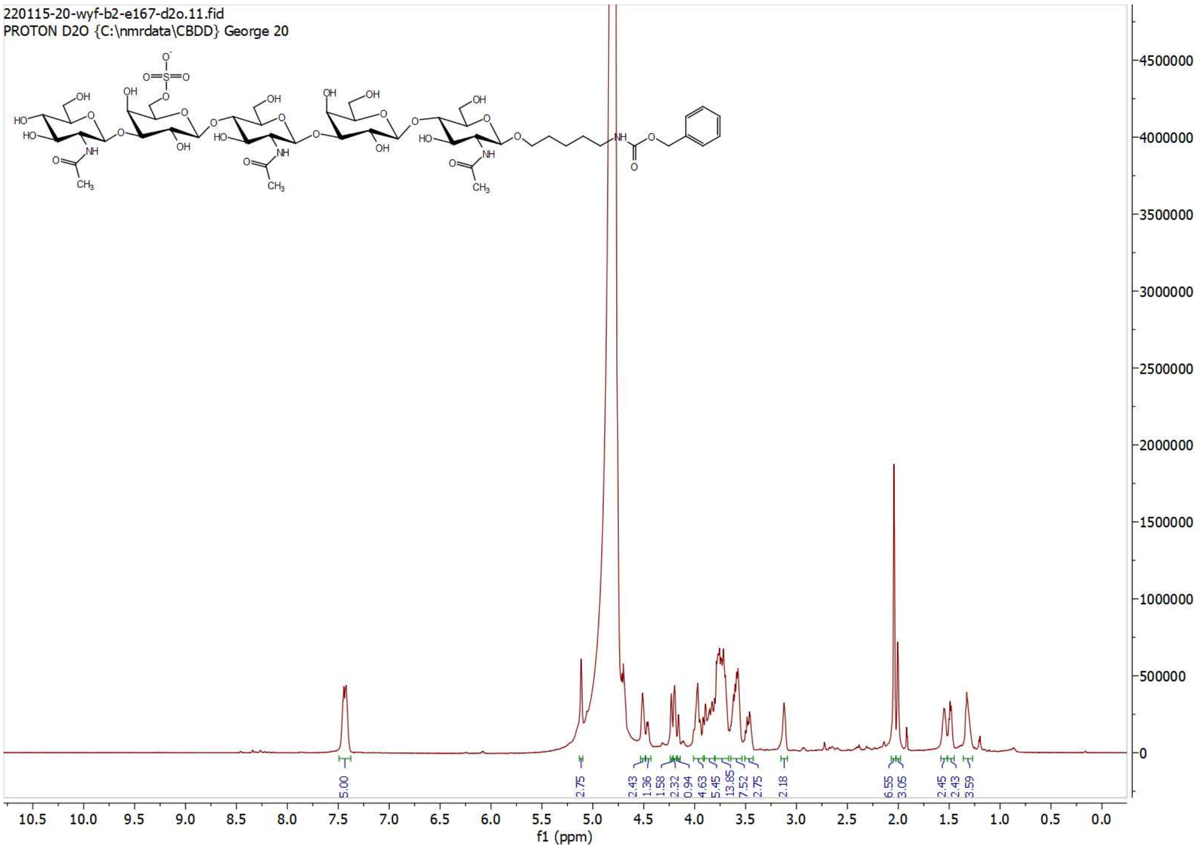

^{1}H NMR of 15; 600MHz; D_2O

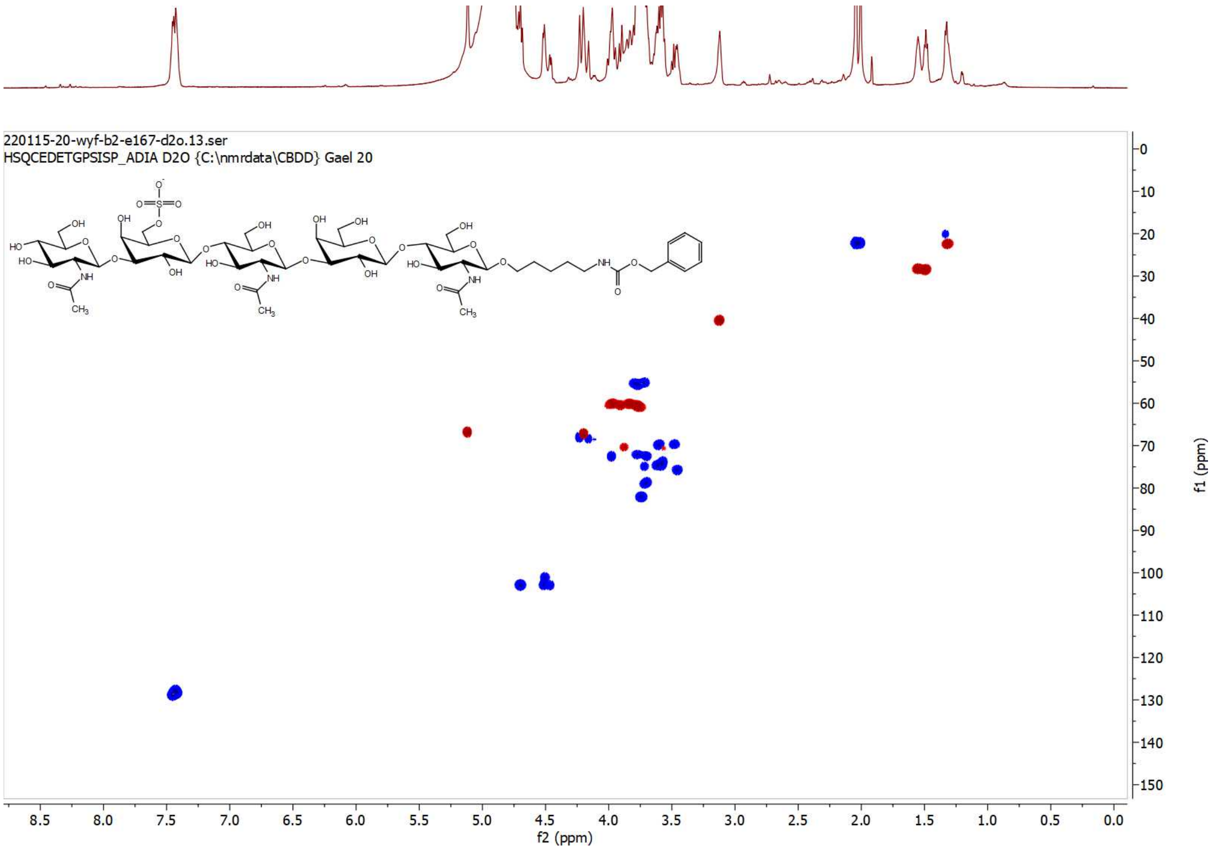

HSQC of 15; 600 MHz/150 MHz, D_2O

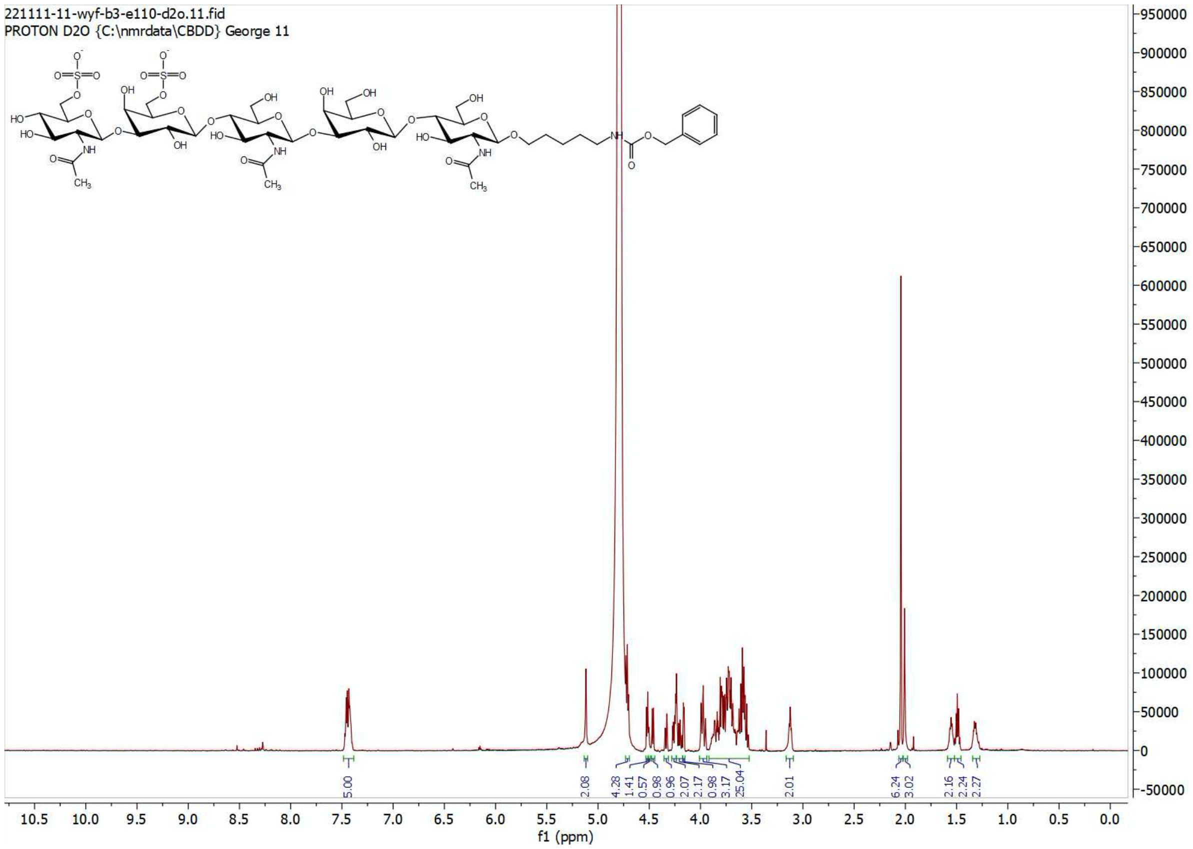

^{1}H NMR of 16; 600MHz; D_2O

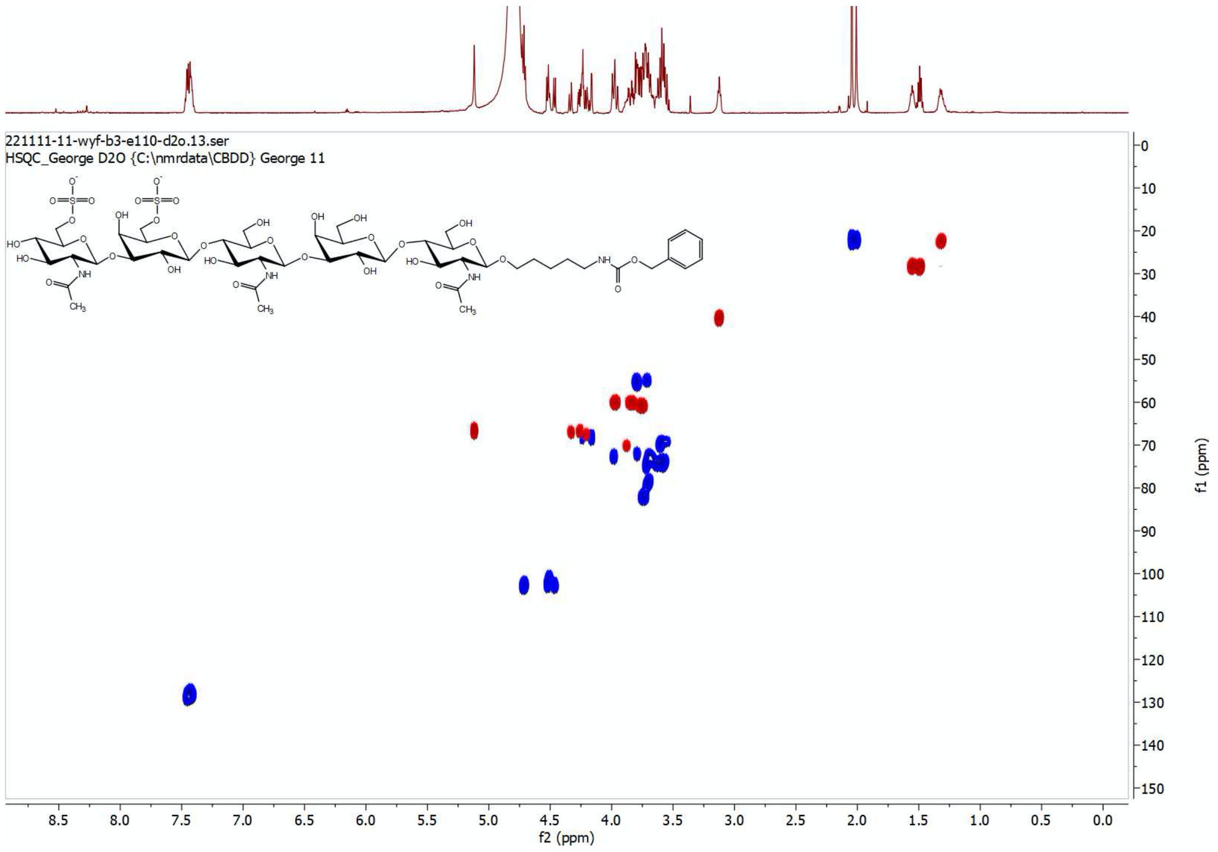

HSQC of 16; 600 MHz/150 MHz, D_2O

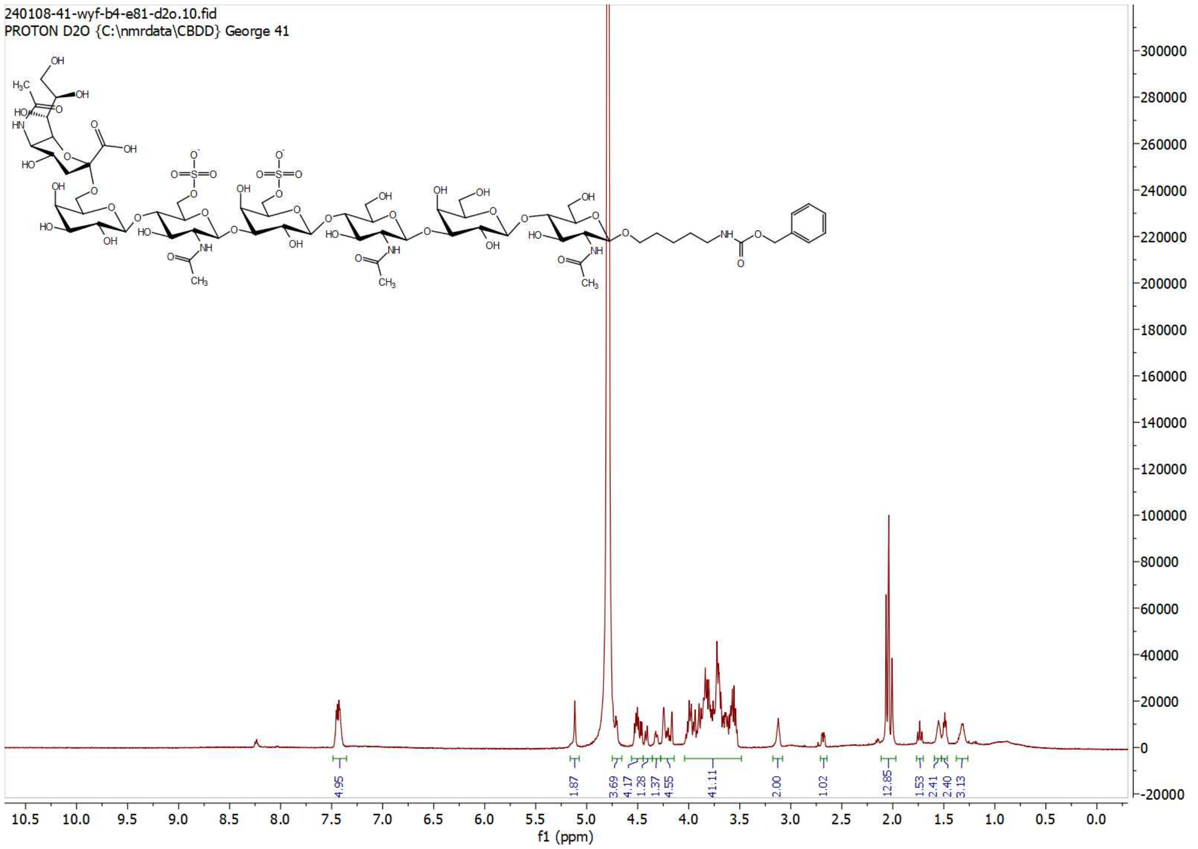

^{1}H NMR of 18; 600MHz; D_2O

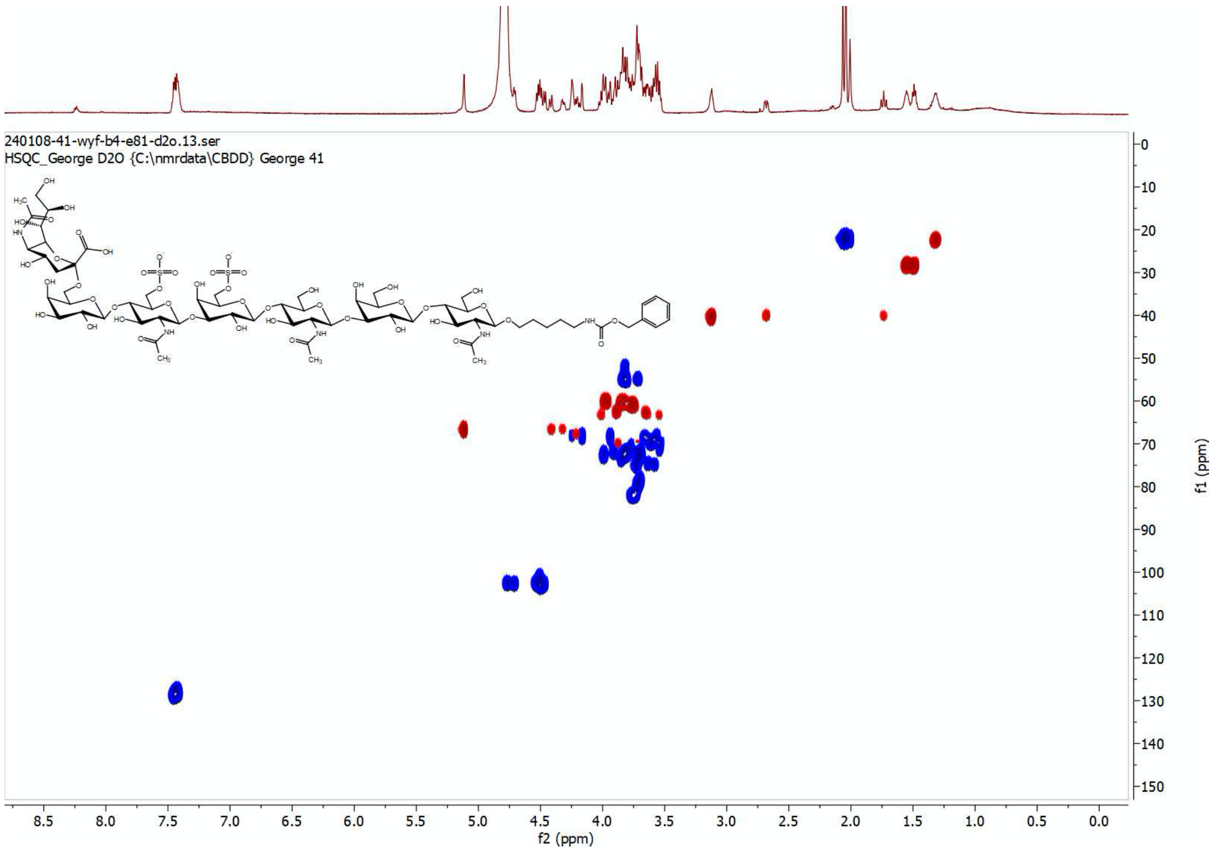

HSQC of 18; 600 MHz/150 MHz, D_2O

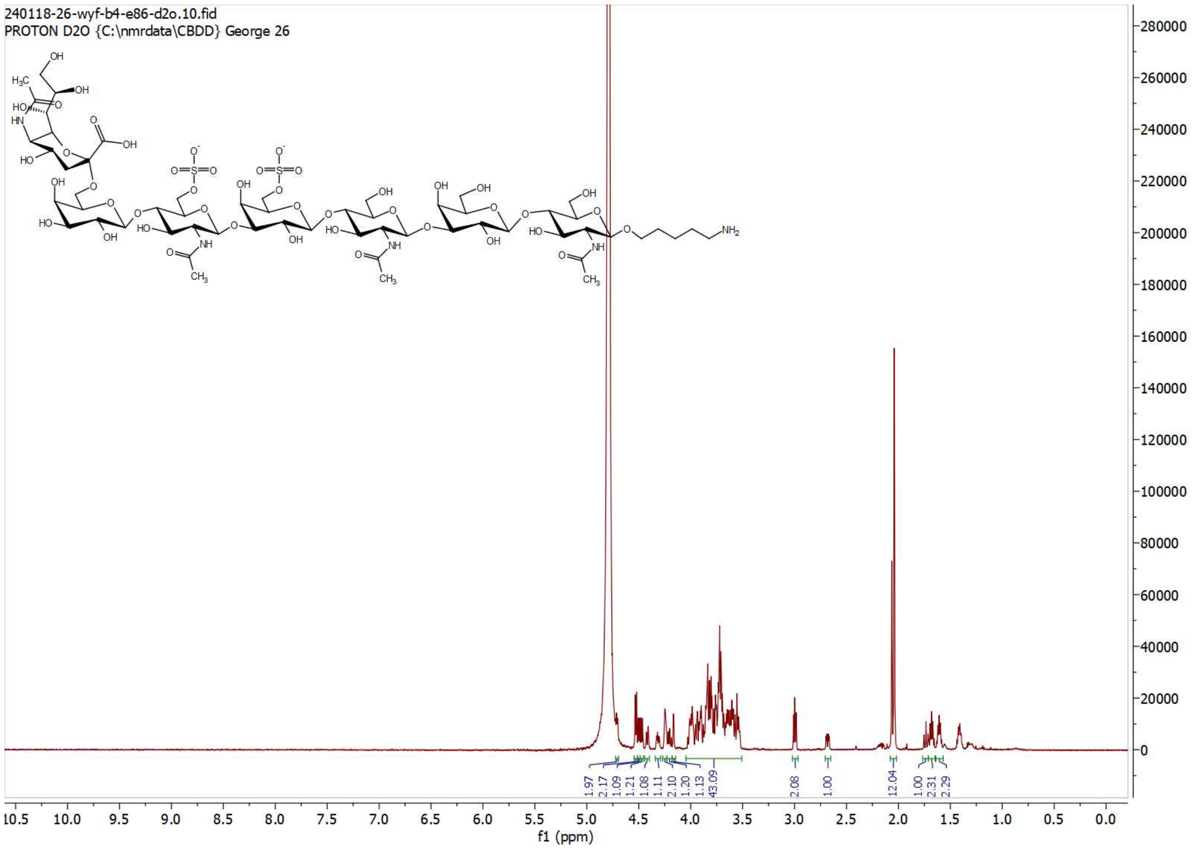

^{1}H NMR of 19; 600MHz; D$_2$O

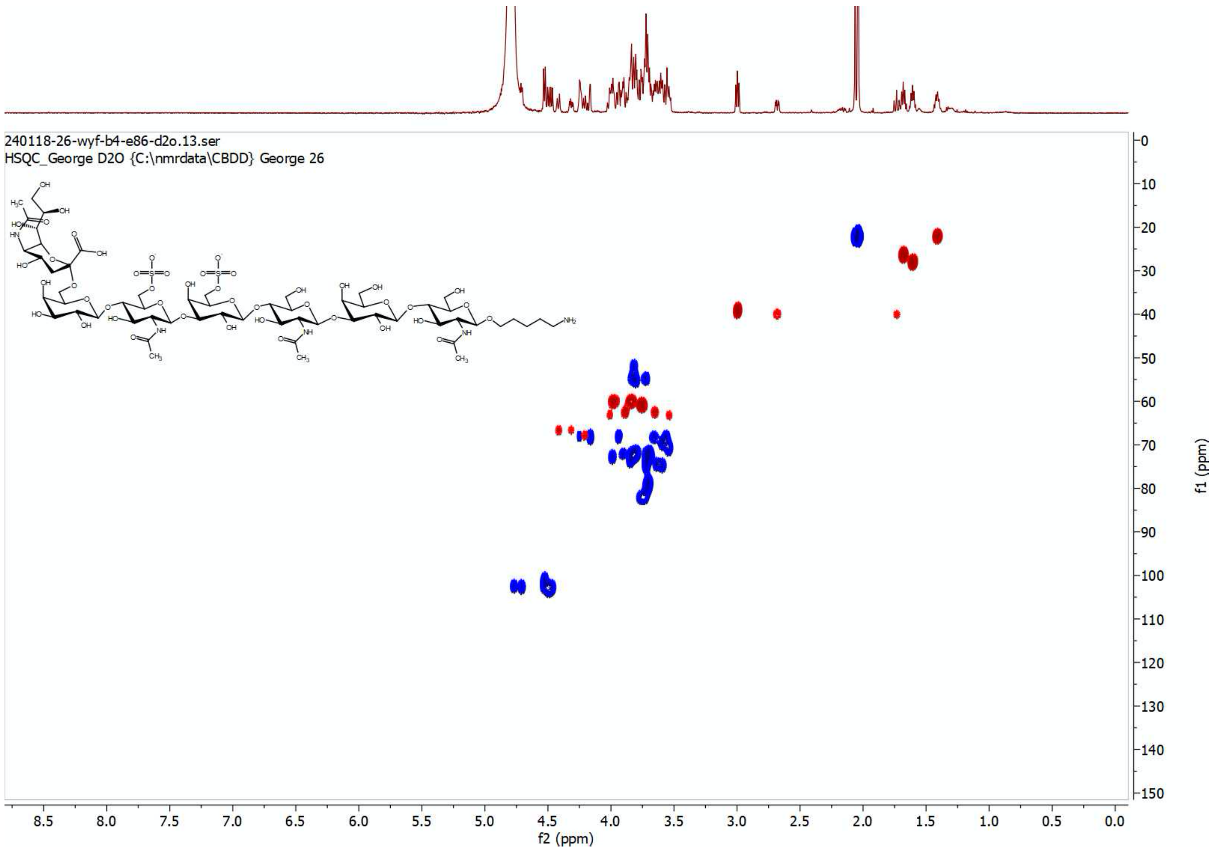

HSQC of 19; 600 MHz/150 MHz, D$_2$O

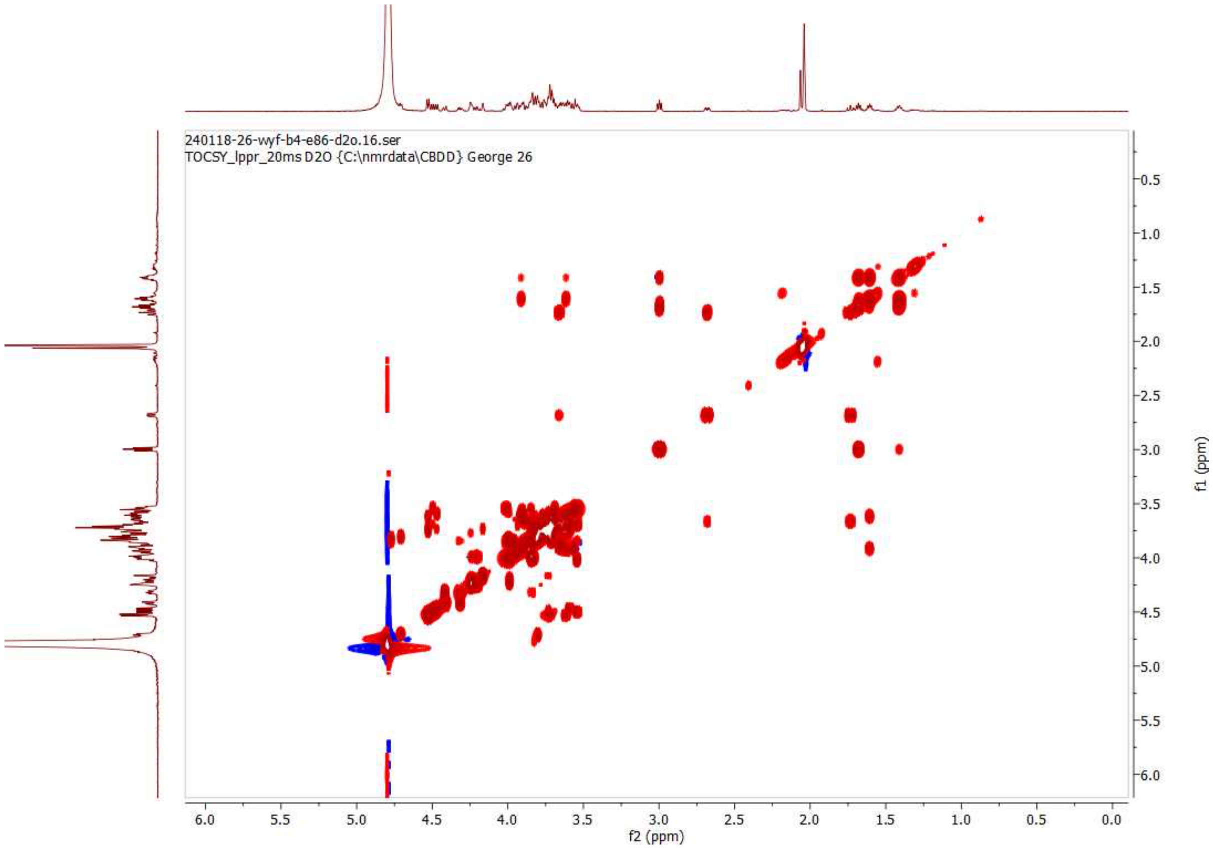

TOCSY (20 ms) of 19; 600MHz; D_2O

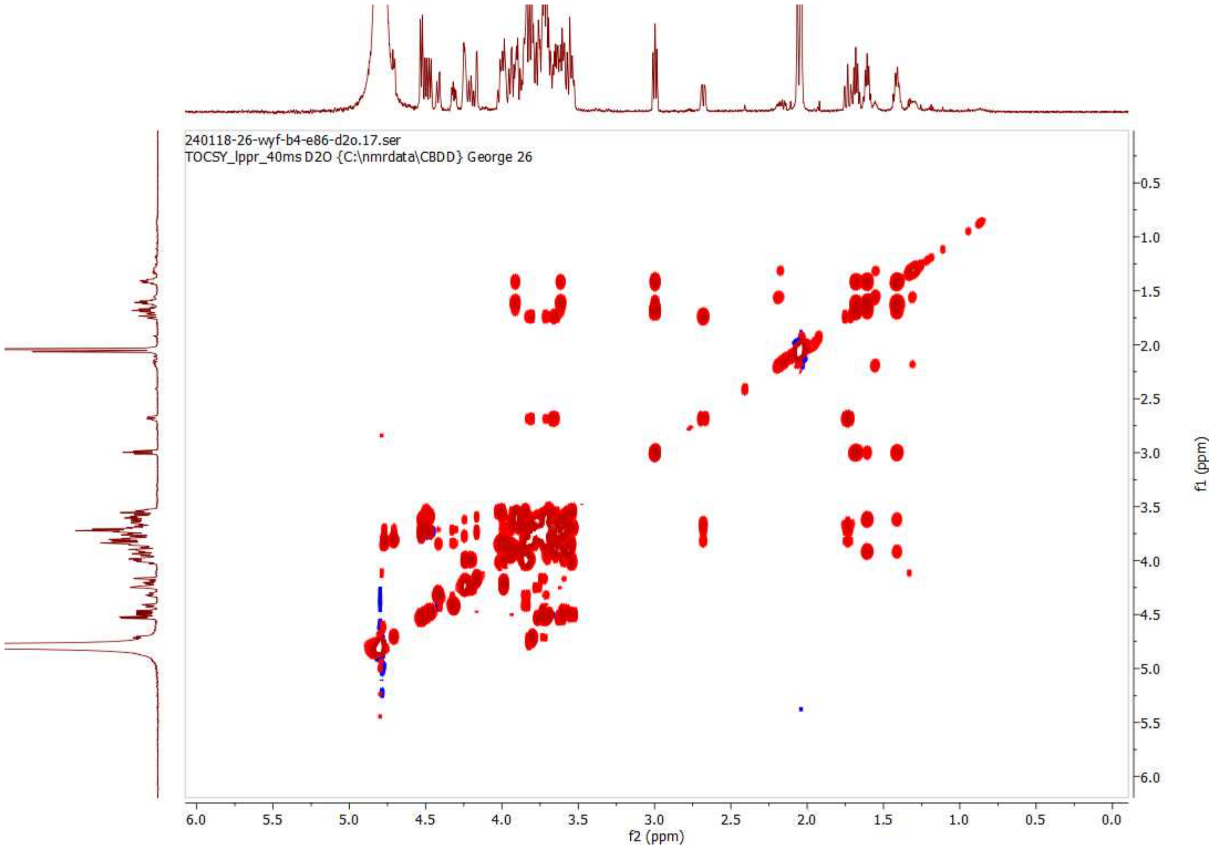

TOCSY (40 ms) of 19; 600MHz; D_2O

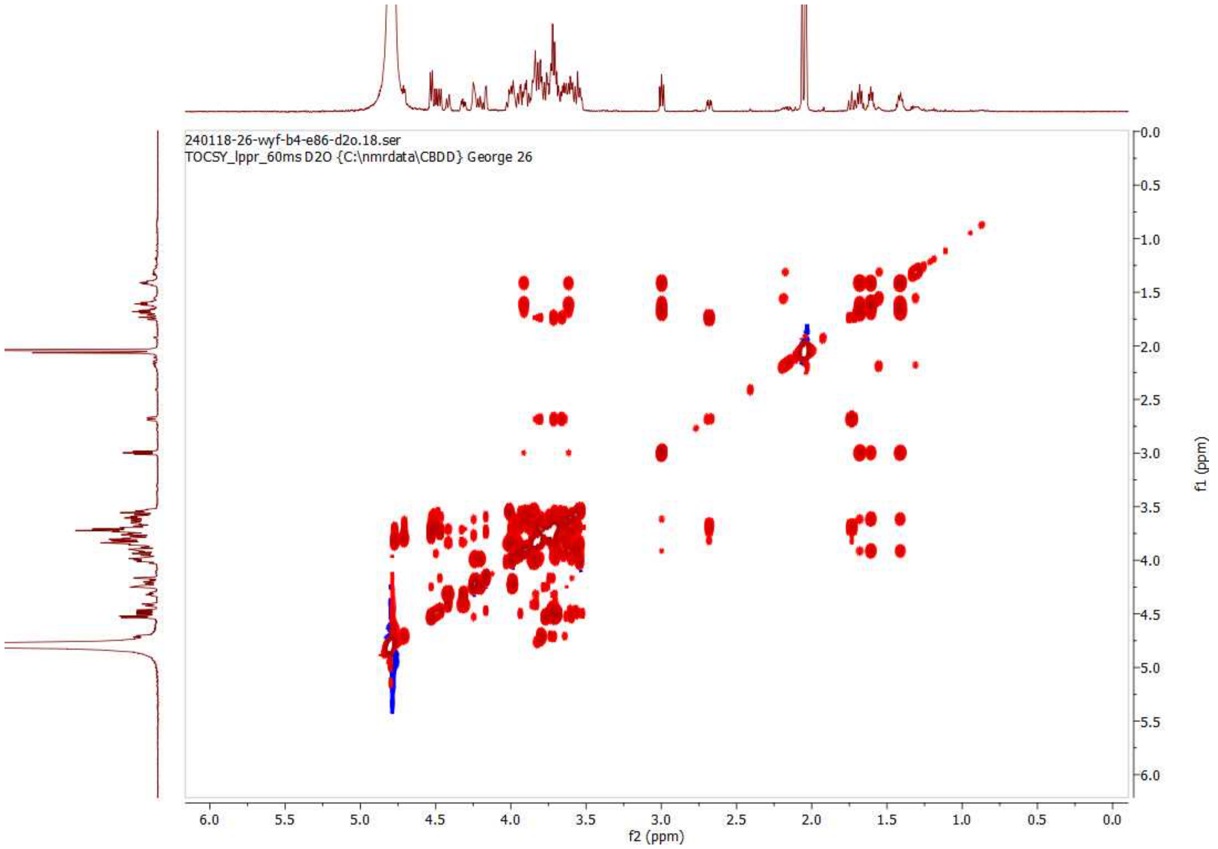

TOCSY (60 ms) of 19; 600MHz; D_2O

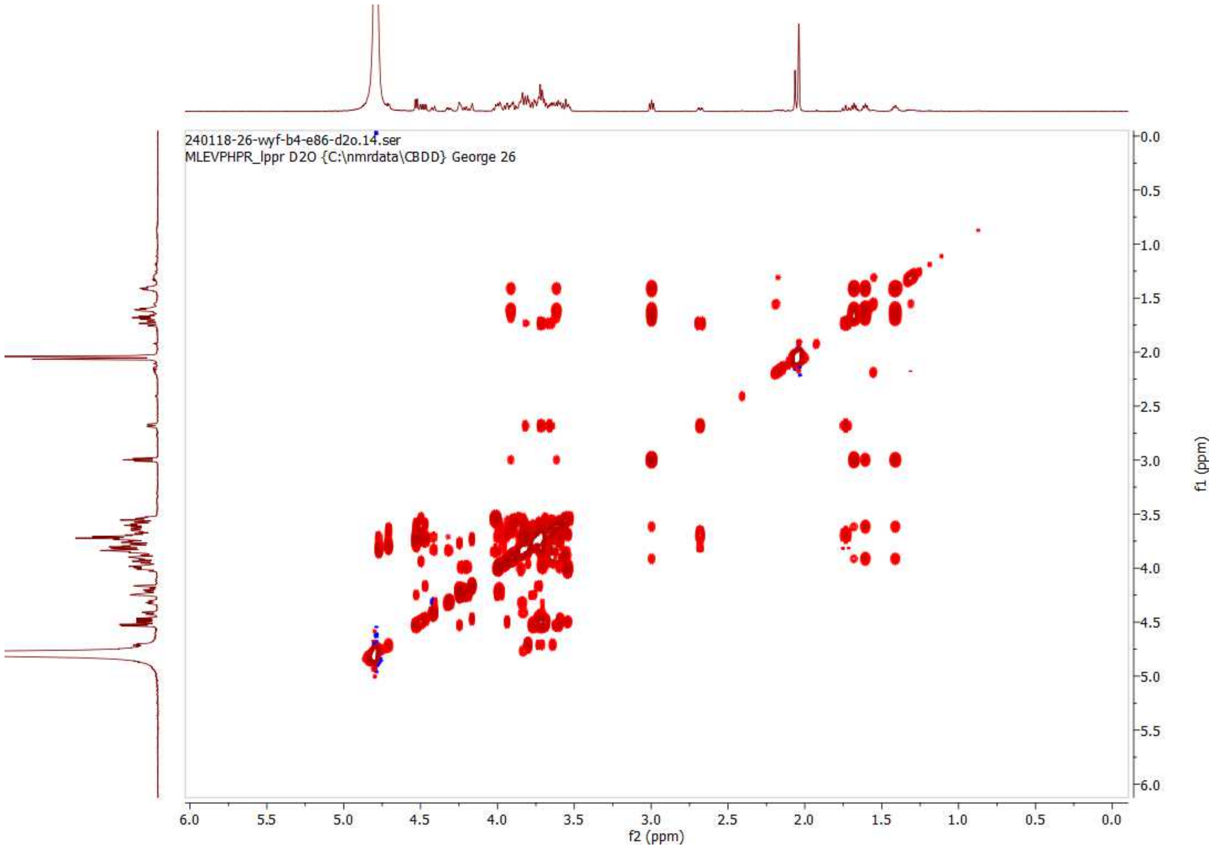

TOCSY (80 ms) of 19; 600MHz; D_2O

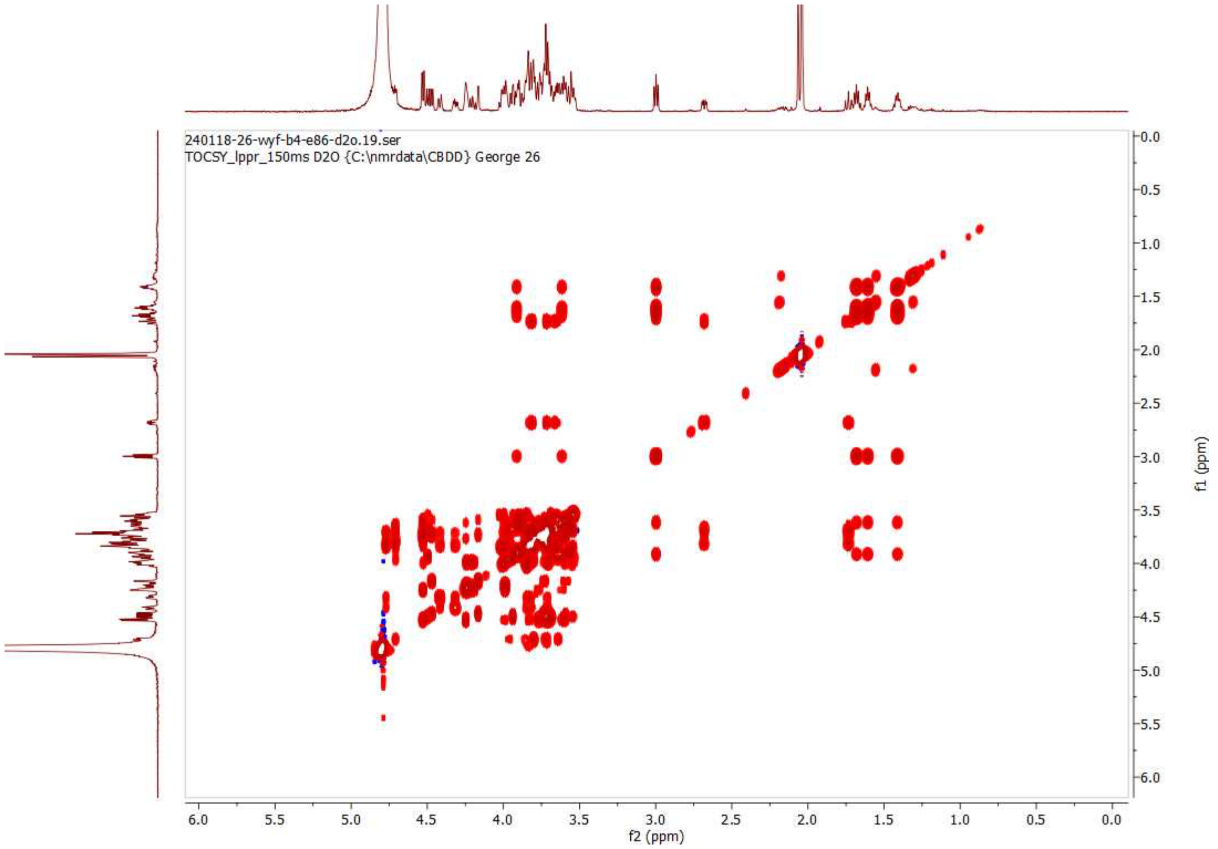

TOCSY (150 ms) of 19; 600MHz; D_2O
